# FLOWR: flow matching for structure-aware de novo, interaction- and fragment-based ligand generation

**DOI:** 10.1038/s43588-026-00998-8

**Published:** 2026-05-28

**Authors:** Julian Cremer, Ross Irwin, Alessandro Tibo, Jon Paul Janet, Simon Olsson, Djork-Arné Clevert

**Affiliations:** 1 Machine Learning and Computational Sciences, Pfizer Worldwide R&D, Berlin, Germany; 2 Molecular AI, Discovery Sciences, R&D, AstraZeneca, Gothenburg, Sweden; 3 Department of Computer Science and Engineering, Chalmers University of Technology and University of Gothenburg, Gothenburg, Sweden

**Keywords:** Cheminformatics, Structure-based drug design, Drug discovery and development, Lead optimization

## Abstract

Here we introduce FLOWR, a structure-based framework for the generation and optimization of three-dimensional ligands. FLOWR integrates continuous and categorical flow matching with equivariant optimal transport, enhanced by an efficient protein pocket conditioning. Alongside FLOWR, we present SPINDR, a curated dataset comprising ligand–pocket cocrystal complexes specifically designed to address existing data quality issues. Empirical evaluations demonstrate that FLOWR surpasses current state-of-the-art diffusion- and flow-based methods in terms of PoseBusters-validity, pose accuracy and interaction recovery, while offering an inference speed-up, achieving up to 70-fold faster performance. In addition, we introduce FLOWR.MULTI, a highly accurate multi-purpose model allowing for the targeted sampling of ligands that adhere to predefined interaction profiles and chemical substructures for fragment-based design without the need of retraining or any resampling strategies. Collectively, our results indicate that FLOWR and FLOWR.MULTI represent an advancement in artificial intelligence-driven structure-based drug design, substantially enhancing the reliability and applicability of de novo, interaction- and fragment-based ligand generation in real-world drug discovery settings.

## Abstract (web summary)

FLOWR combines continuous and categorical flow matching with equivariant optimal transport and a separate protein pocket encoder to improve validity, pose accuracy and interaction recovery at substantially faster inference speeds.

## Main

Structure-based drug discovery (SBDD) is an integrated computational and experimental approach that leverages the three-dimensional structures of biological macromolecules to guide the rational design and optimization of bioactive compounds. By analyzing protein or nucleic acid binding sites, SBDD aims to identify ligands capable of effectively modulating biological functions^1,2^. Commonly employed techniques within this paradigm include molecular docking, virtual screening and structure-guided ligand optimization^3,4^. Despite notable successes, traditional SBDD methods face substantial challenges, such as the inherent complexity of molecular interactions, the vastness of chemical space and difficulties in accurately predicting ligand binding poses and affinities^5,6^.

Recent advances in deep learning have provided promising avenues to overcome these limitations. Classical computational methods, including molecular docking and virtual screening, typically rely on simplified approximations of molecular interactions and struggle to efficiently explore extensive chemical spaces. By contrast, data-driven deep learning approaches, particularly generative models, have demonstrated potential in capturing complex relationships inherent in distributions of experimentally determined ligand–protein complexes^7–9^.

Among generative modeling techniques, diffusion models have emerged as particularly promising tools for de novo ligand design. These models employ iterative stochastic processes to progressively refine molecular structures from initial random noise into chemically valid conformations^10–12^. By incorporating pocket-specific constraints during generation, diffusion models effectively capture the geometric and chemical subtleties of protein–ligand interactions, addressing the challenge of accurately predicting binding poses while generating diverse sets of ligands^12–17^.

Nevertheless, existing diffusion-based approaches are not without drawbacks. Their reliance on iterative stochastic sampling can result in molecules exhibiting strained conformations, uncommon substructures and reduced drug-likeness^17^. In addition, these methods typically suffer from prolonged sampling times compared to alternative generative frameworks^18^.

Recently, generative flow matching models have emerged as an alternative paradigm, offering substantial improvements in generation efficiency^19^. Notably, flow matching approaches employing mini-batch^20^ and equivariant optimal transport^21^ have been proposed, with the latter demonstrating particular efficacy in molecular generation tasks^18^.

Building upon these insights, we introduce FLOWR, a flow matching model specifically designed for the de novo generation of three-dimensional ligands explicitly conditioned on structural constraints. Our framework enables the efficient generation of ligands informed by the geometry of a protein pocket by using a dedicated pocket encoding scheme in contrast to prior works. In addition, we propose FLOWR.MULTI, a versatile extension capable of multi-purpose conditional generation. This model can efficiently and accurately generate ligands adhering to predefined interaction profiles between ligand atoms and pocket residues and can design ligands around specific chemical substructures such as scaffolds and functional groups, facilitating scaffold elaboration, scaffold hopping and fragment-based ligand design—all without requiring model retraining or computationally expensive stabilization techniques during inference as in prior work^16^.

However, the evaluation of SBDD methodologies remains challenging, primarily due to inherent data quality concerns and prevalent data leakage issues in widely utilized benchmark datasets^22,23^. In particular, the commonly employed CROSSDOCKED2020 dataset^24^ exhibits substantial limitations for practical drug discovery applications, stemming from its reliance on rigid-pocket cross-docking protocols. Consequently, ligands are artificially constrained into noncognate binding pockets, causing models trained on such data to internalize biased, flawed and unrealistic distributions of ligand–pocket interactions.

To address these critical issues, we introduce SPINDR, a high-quality benchmark dataset specifically developed for SBDD, derived from the recently presented PLINDER dataset^23^. In constructing SPINDR, we implemented an extensive filtering and structural refinement pipeline designed to correct structural defects prevalent in existing datasets^25^, accurately infer protonation states and atomic-resolution protein–ligand interaction profiles, and minimize potential data leakage between training and test sets by maintaining the PLINDER dataset split.

In summary, we propose the FLOWR model that improves upon existing approaches in both generative quality and computational efficiency. Moreover, our multi-purpose approach, FLOWR.MULTI, enables the generation of ligands conditioned on specific interaction profiles or chemical substructures, substantially increasing the proportion of ligands closely aligned with reference complexes and enhancing applicability in downstream tasks such as hit expansion, hit-to-lead and lead optimization. Finally, our SPINDR dataset provides a high-quality resource for training and evaluating three-dimensional (3D) generative models, addressing limitations—particularly regarding pose quality and data leakage—in currently available datasets.

## Results

**Fig. 1:**
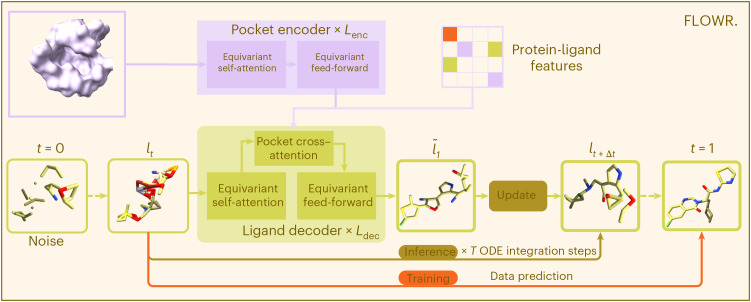
Overview of FLOWR. A schematic overview of the FLOWR model for 3D ligand generation. A protein pocket is encoded and passed, along with the noisy ligand *l*_*t*_, into the ligand decoder, which is trained to produce a denoised ligand ${\widetilde{l}}_{t}$. Optionally, a set of desired pocket–ligand features can be incorporated. A mixed continuous and categorical flow matching integration scheme is then used to push *l*_*t*_ toward the data distribution and generate a sample ${\widetilde{l}}_{1}$ by solving the probability-flow ordinary differential equation (ODE) from *t* = 0 to *t* = 1 with a forward Euler solver using *T* uniform steps. The FLOWR model takes as input pocket coordinates along with atom, bond and residue types, as well as ligand coordinates (with added noise), atom types and bond types. Pocket features are processed through *L*_enc_ sequential blocks consisting of equivariant self-attention and equivariant feed-forward layers, resulting in a pocket encoding. This pocket encoding is subsequently integrated via equivariant cross-attention into *L*_dec_ blocks of equivariant self-attention that process ligand features. Finally, FLOWR outputs denoised ligand coordinates, atom types, bond types and charges. During inference, the pocket encoding is computed only once and reused for all ligand generation steps. Atom colors: C (yellow), N (blue), O (red), Cl (green).

We present FLOWR—a flow-based generative model for de novo ligand generation conditioned on a protein pocket and desired pocket–ligand features. We assume access to a dataset containing tuples of a ligand *l*, a protein pocket ${\mathcal{P}}$ to which the ligand binds and optionally a matrix ${\mathcal{I}}\in {{{{\mathbb{N}}}}}^{M\times N}$ of atomic protein–ligand features, where *M* and *N* refer to the number of atoms in the protein and ligand, respectively. In Fig. 1, we show an overview of how our model generates ligands based on protein pocket and pocket–ligand feature conditioning.

The neural network architecture for FLOWR is based on the recently proposed SEMLA architecture^18^, an *E*(3)-equivariant message passing framework with latent attention that achieves state-of-the-art results on unconditional 3D molecular generation. We extend SEMLA to allow conditional generation by incorporating a separate pocket encoder and adding an equivariant cross-attention module within the ligand decoder, enabling structural conditioning on the protein pocket and desired protein–ligand features. Critically, the pocket encoder does not depend on the flow time *t* or the noisy ligand *l*_*t*_, meaning only one forward pass through the encoder is required when generating ligands, amortizing the encoding cost over many samples. We further improve the base architecture by introducing a gated equivariant feed-forward module and passing bond embeddings into every self-attention layer, yielding improved validity and efficiency. Full architectural details and hyperparameters are provided in the Methods.

FLOWR jointly models continuous (coordinates) and discrete (atom types and bond orders) molecular features using a combination of continuous flow matching^19,20^ for coordinates and discrete flow models^26^ for categorical properties, with equivariant optimal transport^21^ to reduce transport costs. Ligand formal charges are directly predicted. The model learns to recover the clean ligand *l*_1_ from a noisy interpolant *l*_*t*_ via ${p}_{1| t}^{\theta }({l}_{1}| {l}_{t},t;{\mathcal{P}},{\mathcal{I}})$, minimizing mean-squared error for coordinates and cross-entropy for categorical features. Given ${\mathcal{P}}$ and optionally ${\mathcal{I}}$, ligands are generated by iteratively refining an initial noisy ligand *l*_0_ ≈ *p*_noise_ through Euler integration of the learned vector field. Full training and sampling details are provided in the ‘Training and inference’ section in the Methods.

We extend the FLOWR model with FLOWR.MULTI—a multi-purpose training and inference framework that simultaneously supports both de novo generation and any form of fragment-based sampling, similar to scaffold hopping, scaffold elaboration, fragment linking and fragment-based generation, which is highly relevant from hit expansion to lead optimization campaigns. As before, we consider a protein pocket ${\mathcal{P}}$ and a protein–ligand feature matrix ${\mathcal{I}}$, while assuming a (set of) predefined fragmentation(s) applied onto a ligand *l*. Let the ligand consist of *N* atoms with ${{\bf{l}}}_{{\bf{1}}}\in {{\mathbb{R}}}^{N\times 3}$ denoting its coordinates, and, for simplicity, assume that it is split into two fragments containing *n*_1_ and *n*_2_ atoms, respectively, with *n*_1_ + *n*_2_ = *N*. Thus, we have $${{\bf{l}}}_{{{\bf{t}}}_{1}{{\bf{t}}}_{{\bf{2}}}}=\left(\begin{array}{l}{{\bf{l}}}_{{{\bf{t}}}_{{\bf{1}}}}^{1}\\ {{\bf{l}}}_{{{\bf{t}}}_{{\bf{2}}}}^{2}\end{array}\right)\in {{\mathbb{R}}}^{({n}_{1}+{n}_{2})\times 3}$$ with *t*_1_ and *t*_2_ sampled independently from a uniform distribution. Setting $${{\bf{t}}}_{{\bf{12}}}=\left(\begin{array}{l}{t}_{1}\\ {t}_{2}\end{array}\right)$$, the linear interpolation reads $${{\bf{l}}}_{{{\bf{t}}}_{1}{{\bf{t}}}_{{\bf{2}}}}={{\bf{t}}}_{{\bf{12}}}\odot {{\bf{l}}}_{{\bf{1}}}+({\bf{1}}-{{\bf{t}}}_{{\bf{12}}})\odot {{\bf{l}}}_{{\bf{00}}}=\left(\begin{array}{l}{t}_{1} {{\bf{l}}}_{{{\bf{t}}}_{{\bf{1}}}}^{{\bf{1}}}+(1-{t}_{1}) {{\bf{l}}}_{{\bf{0}}}^{{\bf{1}}}\\ {t}_{2} {{\bf{l}}}_{{{\bf{t}}}_{{\bf{2}}}}^{2}+(1-{t}_{2}) {{\bf{l}}}_{{\bf{0}}}^{{\bf{2}}}\end{array}\right)$$, where ⊙ denotes elementwise multiplication, **1** is the all-ones vector and **l**_**00**_ ≈ *p*_noise_ is the initial noise sample.

The goal is to learn a joint probability distribution $${p}_{1| {t}_{1}{t}_{2}}^{\theta }({{\bf{l}}}_{{\bf{1}}}| {{\bf{l}}}_{{{\bf{t}}}_{1}{{\bf{t}}}_{2}},$$$${{\bf{t}}}_{{\bf{12}}};{\mathcal{P}},{\mathcal{I}})$$, from which at inference we sample $${\widetilde{{\bf{l}}}}_{1}=\left(\begin{array}{l}{\widetilde{{\bf{l}}}}_{1}^{1}\\ {\widetilde{{\bf{l}}}}_{1}^{2}\end{array}\right)\in {{\mathbb{R}}}^{N\times 3}$$ to retrieve the joint vector field $$\begin{array}{c}f({{\bf{l}}}_{{{\bf{t}}}_{1}{{\bf{t}}}_{2}},\end{array}$$$$\begin{array}{l}{{\bf{t}}}_{{\bf{12}}};{\mathcal{P}},\end{array}{\mathcal{I}})={\mathop{{\bf{l}}}\limits^{ \sim }}_{1}-{{\bf{l}}}_{{\bf{00}}}$$.

Denoting the per-fragment step sizes by Δ*t*_1_ = *t*_1_ + *s*_1_ and Δ*t*_2_ = *t*_2_ + *s*_2_, where *s*_*i*_ is derived from the number of inference steps, and defining $$\Delta {t}_{12}=\left(\begin{array}{l}\Delta {t}_{1}\\ \Delta {t}_{2}\end{array}\right)$$, the Euler update step reads

1 $${{\bf{l}}}_{{{\bf{t}}}_{1}+\Delta {{\bf{t}}}_{1}{{\bf{t}}}_{2}+\Delta {{\bf{t}}}_{2}}={{\bf{l}}}_{{{\bf{t}}}_{1}{{\bf{t}}}_{{\bf{2}}}}+\Delta {t}_{12}\odot f({{\bf{l}}}_{{{\bf{t}}}_{1}{{\bf{t}}}_{{\bf{2}}}},{{\bf{t}}}_{{\bf{12}}};{\mathcal{P}},{\mathcal{I}})=\left(\begin{array}{l}{{\bf{l}}}_{{{\bf{t}}}_{{\bf{1}}}}^{{\bf{1}}}+\Delta {t}_{1}\cdot ({\widetilde{{\bf{l}}}}_{1}^{1}-{{\bf{l}}}_{{\bf{0}}}^{{\bf{1}}})\\ {{\bf{l}}}_{{{\bf{t}}}_{{\bf{2}}}}^{{\bf{2}}}+\Delta {t}_{2}\cdot ({\widetilde{{\bf{l}}}}_{1}^{2}-{{\bf{l}}}_{{\bf{0}}}^{{\bf{2}}})\end{array}\right).$$

Notably, when setting, for example, *t*_1_ = 1 and ${{\bf{l}}}_{{\bf{0}}}^{1}={{\bf{l}}}_{{{\bf{t}}}_{{\bf{1}}}}^{1}={{\bf{l}}}_{{\bf{1}}}^{1}$, we have Δ*t*_1_ = 1, as *s*_1_ becomes 0 and the update becomes

2 $${{\bf{l}}}_{1{{\bf{t}}}_{2}+\Delta {{\bf{t}}}_{2}}=\left(\begin{array}{l}{\widetilde{{\bf{l}}}}_{1}^{1}\\ {{\bf{l}}}_{{{\bf{t}}}_{{\bf{2}}}}^{2}+\Delta {t}_{2}\cdot ({\widetilde{{\bf{l}}}}_{1}^{2}-{{\bf{l}}}_{{\bf{0}}}^{2})\end{array}\right).$$

In this scenario, the atoms corresponding to *t*_1_ = 1 remain fixed to be the predictions of the model at each inference step. Assuming the model has successfully learned the identity mapping ${\widetilde{{\bf{l}}}}_{1}^{1}={{\bf{l}}}_{{\bf{1}}}^{{\bf{1}}}$ for the conditional distribution $${p}_{1| 1t2}^{\theta }({{\bf{l}}}_{{\bf{1}}}| {{\bf{l}}}_{1{{\bf{t}}}_{2}},(\begin{array}{l}1\\ {t}_{2}\end{array});{\mathcal{P}},{\mathcal{I}})$$, this approach effectively resembles the concept of so-called inpainting. Originally proposed in computer vision^27^, inpainting has already been adopted for molecular generation tasks^16^. However, unlike ref. ^16^, which requires costly resampling steps, and ref. ^28^, which suffers from reduced structural fidelity, FLOWR.MULTI avoids both limitations while maintaining or even enhancing quality across all generation modes. Specifically, since FLOWR.MULTI is explicitly trained on a diverse set of inpainting tasks, we anticipate substantial improvements in validity rates, structural accuracy and inference efficiency, considerably broadening its downstream applicability.

Modeling interactions between protein pockets and ligands has recently been gaining attention as a method for evaluating the quality of binding poses and designing better small molecule drug candidates^29,30^. At the same time, questions have been raised about the quality of existing benchmark datasets—PDBBind^31^ has been found to contain covalently bound ligands, missing atoms in pockets and steric clashes between the pocket and the ligand^25^. CROSSDOCKED2020^24^, another commonly used dataset for pocket-conditioned ligand generative models, is based on the PDBBind General set and is also likely to share many of these structural defects. Moreover, questions have also been raised as to how well temporal data splits, which are commonly used to create benchmark test sets, are able to assess models’ abilities to generalize to unseen data, as there are often close structural similarities between complexes in the training and test sets.

To address the issues of data quality and information leakage and to provide rich, fine-grained information on the interactions between protein pockets and small molecule ligands, we present SPINDR (Small molecule Protein Interaction Dataset, Refined). Using the recently proposed PLINDER dataset^23^ as a starting point we apply an extensive filtering and processing pipeline to produce a refined set of high-quality structures. Specifically, to create the SPINDR dataset, we took the PLINDER dataset release June 2024 (PLINDER version 2) and applied the following processing pipeline:

1. Initial filtering: We remove all PLINDER systems which contain more than one ligand or have more than one protein chain in the pocket. We then remove all systems where the ligand is marked as one or more of the following: ‘oligo’, ‘ion’, ‘cofactor’, ‘artifact’, ‘fragment’, ‘covalent’ or ‘other’.
2. Structure refinement: We use Schrodinger protein preparation wizard (which uses the OPLS 2005 forcefield^32^) to refine the structure of the remaining systems. These tools perform the following:
  Add missing atoms to partially filled residues in the protein
  Convert some nonstandard residue types to standard ones
  Assign protonation states to heavy atoms and add hydrogen atoms to both the protein and ligand
  Infer bonds and formal charges for both the protein and ligand
  Apply local energy minimization to the protein–ligand complex
3. Infer protein–ligand interactions: We use ProLIF^33^ to infer the interactions between the protein and ligand at an atomic resolution, creating a binary matrix of shape *N*_prot_ × *N*_lig_ × ∣*S*∣, where *N*_prot_ is the number of atoms in the protein, *N*_lig_ is the number of atoms in the ligand and *S* is the set of possible interaction types. We apply ProLIF with the default settings and infer all supported interaction types, ∣*S*∣ = 13.
4. Quality filtering: We apply a final filtering step and accumulate the processed systems into train, validation and testing splits. Here, we ensure that all systems contain RDKit-valid ligands. We also filter out any system which contains atoms other than {H, C, N, O, F, P, S, Cl, Se, Br} and any system with fewer than five residues in the pocket. In addition, we filter out all systems containing NAG ligands because we found these were highly overrepresented, which would probably create an unwanted bias for generative models. We also filter out all systems derived from the PDB complex ‘1mvm’ because it contains many small DNA fragments and was not originally filtered by PLINDER.
5. Data deduplication: As existing datasets often contain substantial structural redundancy, we experiment with two data deduplication strategies presented in Supplementary Information section 1. In practice, we found little empirical difference between the original and deduplicated datasets, despite their substantially smaller size (10,000–15,000 fewer complexes), suggesting there is a significant amount of redundancy in the original data. However, we use the nondeduplicated version of SPINDR for the remainder of this study, though we make all three versions publicly available to enable further investigation of deduplication strategies in ligand generation tasks.

Our final dataset contains 35,666 protein–ligand complexes, making SPINDR the largest dataset of high-quality, refined structures derived directly from crystallographic data. In Table 1, we compare some of the features of SPINDR to other commonly used dataset for SBDD and docking. Notably, in addition to the features in Table 1, we maintain the same data splits as PLINDER. The PLINDER splits were carefully selected to minimize data leakage between train and test sets and to ensure test systems were always of high quality. This careful curation enables realistic assessment of models’ generalizability to unseen data, unlike many existing benchmarks which contain substantial train-test data leakage^23^.

**Table 1:** Feature comparison of SPINDR with commonly used datasets

Dataset	Crystal structure	Energy minimized	Explicit	Protein–ligand
	Complexes	Conformations	Hydrogens	Interactions
CROSSDOCKED	✗	✗	✗	✗
PDBBIND	*✓*	✗	✗	✗
SPINDR	*✓*	*✓*	*✓*	*✓*

An overview of the additional features provided by the SPINDR dataset compared to datasets commonly used for training generative models for structure-based drug design and docking tasks. The checkmarks indicate presence, and the crosses indicate absence of each feature.

We compare our model against recently published generative methods on the commonly used CROSSDOCKED2020 dataset as an initial benchmark. As can be seen in Extended Data Table 1, the proposed FLOWR model substantially outperforms all baseline methods (POCKET2MOL^14^, DIFFSBDD^16^, TARGETDIFF^15^, DRUGFLOW^34^ and PILOT^17^) across the evaluated metrics on the CROSSDOCKED2020 test dataset. Specifically, FLOWR achieves the highest PoseBusters-validity (PB-validity) (0.92 ± 0.22), lowest strain energy (87.83 ± 74.30), best AutoDock-Vina scores (mean −6.29 ± 1.56, minimized −6.48 ± 1.45) and lowest Wasserstein distances for bond angles (0.96) and bond lengths (0.27). In addition, FLOWR demonstrates substantially faster inference time (12.05 ± 8.01 s) compared to other methods. These results indicate that FLOWR generates ligand conformations closest to the test set distribution and with superior computational efficiency. We note that the second best model, PILOT, also shows substantially better results compared to all other methods, especially in terms of PB-validity (0.83 ± 0.33). Thus, we selected PILOT as our main competitor for the remaining of this study.

**Extended Data Fig. 1:**
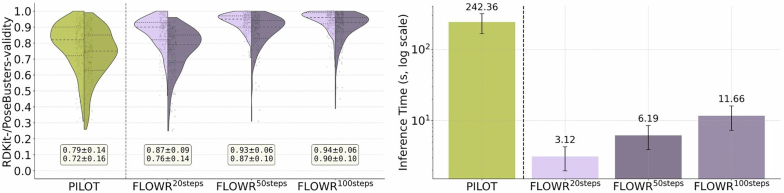
Comparison of PILOT and FLOWR on RDKit-PoseBusters-validity and inference speed on the SPINDR test set. **(Left)** Violin plots depicting the distribution of per-target RDKit validity (left half) and PoseBusters (PB) validity (right half) rates across all 225 targets in the SPINDR test set for each model. Each target’s validity rate is computed as the fraction of valid molecules out of 100 generated ligands. Dashed horizontal lines indicate the lower quartile, median, and upper quartile of the distribution. Individual per-target rates are overlaid as scatter points. Text annotations below each violin report the mean +- standard deviation of per-target validity rates (RDkit, PB). FLOWR is evaluated at 20, 50, and 100 integration steps. Note, both RDKit- and PoseBusters-validity are evaluated on the full set of generated ligands per target. **(Right)** Bar plot comparing mean inference times (in seconds, log scale) for generating 100 ligands per target across models. Bar heights represent the mean wall-clock time, with error bars in black indicating the standard deviation. Exact mean values are annotated above each bar. FLOWR timings are evaluated at 20, 50, and 100 integration steps. All models are evaluated using a single Nvidia H100 GPU. Source data

In Extended Data Fig. 1, we compare PILOT and FLOWR trained on the SPINDR training dataset in terms of RDKit-validity, PB-validity and inference speed on the SPINDR test set. Our results indicate that FLOWR generates ligands with substantially higher validity on average. Although RDKit-validity is a two-dimensional ligand-centric measure, the PoseBusters suite^35^ evaluates ligand conformations using well-established 3D ligand–pocket-based metrics, providing a more comprehensive assessment of pose accuracy. FLOWR achieves a substantial improvement over PILOT in both metrics, with an average RDKit-validity of 0.94 ± 0.24 versus 0.79 ± 0.39 and an average PB-validity of 0.88 ± 0.21 versus 0.71 ± 0.18, respectively. Notably, FLOWR substantially improves inference speed, outperforming PILOT by a factor of 20 when using 100 inference steps, as shown in Extended Data Fig. 1, right. This efficiency gain is primarily attributed to FLOWR’s model architecture and the protein pocket encoder requiring only a single forward when integrating the vector field. By contrast, prior models^12,15–17^ often recompute protein pocket embeddings at every sampling step. Notably, the number of integration steps can be reduced as low as 20, achieving a 70-fold speed-up over PILOT while impacting model performance comparably little.

In Extended Data Table 2, we compare PILOT and FLOWR in terms of strain energy (calculated using GenBench3D^36^), AutoDock-Vina score (used as an approximate measure of pose quality and binding affinity^37^) and their ability to generalize to the test set distribution based on Wasserstein distance measures for bond angles, bond lengths and dihedral angles following^11,12,17^. A more comprehensive overview of results is given in Supplementary Information section 2. As flow matching allows for setting the number of inference steps, we also report the same results for different number of steps, namely 20, 50 and 100 (default).

**Extended Data Fig. 2:**
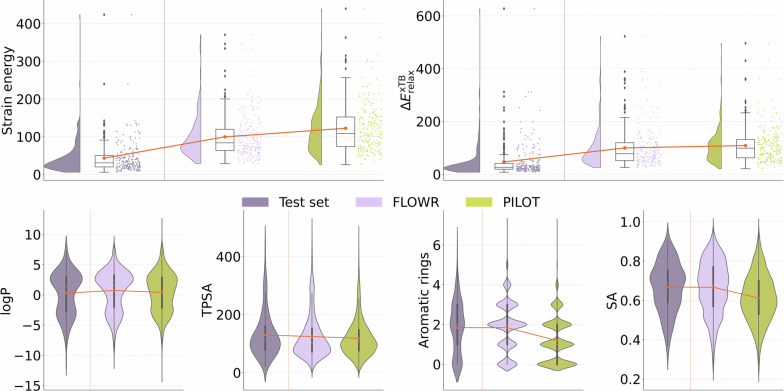
Comparison of PILOT and FLOWR on molecular properties on SPINDR. **(Top left)** Raincloud distribution and box-and-whisker plot showing the distribution of per-target mean strain energy across SPINDR test set targets and 100 sampled ligands per target; red connected dots indicate per-model means. **(Top right)** Raincloud distribution and box-and-whisker plot showing xTB relaxation energies (${E}_{\,{\rm{relax}}}^{{\rm{xTB}}}$, kcal/mol) using GFN2-xTB with implicit solvation and the Analytical Linearized Poisson–Boltzmann (ALPB) solvation model; red connected dots indicate per-model means. Per-target values are computed by averaging over all ligands generated for each target. **(Bottom row)** Violin plots of per-ligand molecular property distributions for octanol–water partition coefficient (logP), topological polar surface area (TPSA), number of aromatic rings, and synthetic accessibility (SA) score; red connected dots indicate per-model means. Across all panels, test set distributions are shown in purple, FLOWR in pink, and PILOT in green. In all box-and-whisker plots, the centre line represents the median, box bounds represent the 25th and 75th percentiles (interquartile range, IQR), and whiskers extend to 1.5x IQR from the box bounds. Data are presented as mean values +- standard deviation. Source data

In terms of strain energy values, FLOWR substantially outperforms PILOT (90.05 ± 52.18 versus 120 ± 71.61). However, we note that, on average, the strain energies of generated ligands do not align well with those of the test set (43.27 ± 41.85), as illustrated in Extended Data Fig. 2, top left. We hypothesize that this discrepancy arises primarily from limited coverage of chemical and conformational space in the training data, due to the relatively low availability of cocrystal structures. Notably, these elevated strain energies reflect subtle deviations in bond angles and lengths rather than gross structural defects-simple MMFF94s-based relaxation with fixed protein pockets reduces strain energies to 28.05 ± 28.25 kcal mol^−1^, below the test set average. In addition, a mean r.m.s.d. of 0.775 ± 0.144 Å; confirms that generated molecules readily relax to low-energy conformations with minimal structural perturbation (Supplementary Fig. 3), whereas mean PB-validity increases to 0.95 ± 0.08 and mean Vina score decreases to −6.97 ± 0.89. In addition, Extended Data Fig. 2, top right, shows the relaxation energy distribution of generated ligands calculated using the GFN2-XTB method^38^ with implicit solvation using the ALPB model^39^ (46.37 ± 64.05 kcal mol^−1^ on the test set; 100.37 ± 59.85 for FLOWR versus 107.89 ± 66.37 for PILOT). Another commonly reported metric in this context is the clash count between ligand and pocket atoms. Using PoseCheck^30^, we observe a clear improvement for FLOWR (5.25 ± 2.21) compared to the PILOT model (6.28 ± 2.61), with FLOWR more closely resembling the test set distribution (4.24).

**Extended Data Fig. 3:**
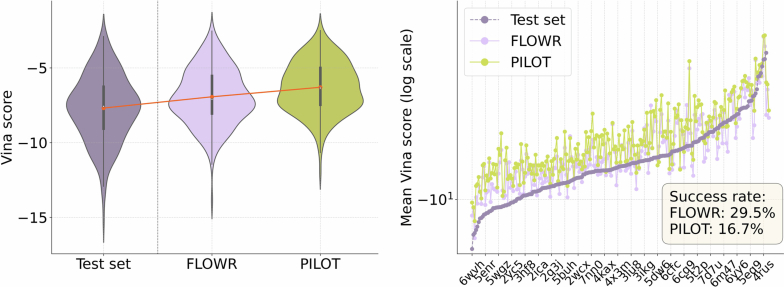
Comparison of PILOT and FLOWR on AutoDock-Vina scores on SPINDR. **(Left)** Violin plots of per-target mean Vina scores across the SPINDR test set targets sampling 100 ligands per target with embedded box plots; red connected dots indicate per-model means. In box plots, the center line represents the median, box bounds represent the 25th and 75th percentiles (interquartile range, IQR), and whiskers extend to 1.5x IQR from the box bounds. **(Right)** Per-target mean Vina scores (symmetric log scale) sorted in ascending order by test set score (purple), with targets labeled every 10th position along the x-axis. FLOWR sample scores are denoted by pink, PILOT scores by green scatter points. The inset text box reports the mean per-target success rate, defined as the fraction of generated ligands achieving a Vina score equal to or lower than the corresponding test set mean. Source data

FLOWR outperforms PILOT in docking assessments, suggesting a higher pose accuracy (−6.93 ± 0.92 versus −6.30 ± 0.96). We not only use Vina’s scoring function with no redocking applied but also report the minimized Vina score, where local energy minimization is applied to the ligand (−7.22 ± 0.92 versus −6.68 ± 1.07). In Extended Data Fig. 3, left, we compare the Vina score distribution across targets and the mean Vina score per target (Extended Data Fig. 3, right, log scale). As can be seen, FLOWR shows a 12.8% increase in success rate (number of ligands per target that are either equal or better than the reference with respect to Vina scoring) with an average success of 29.5%.

Moreover, we measure distribution learning capabilities in terms of bond angle and bond length Wasserstein distances to the test set. Here, FLOWR demonstrates substantially better generalization compared to PILOT with a mean bond angles distance of 1.08 versus 1.82, mean bond lengths distance of 0.35 versus 0.42 and mean dihedral angles distribution distance of 5.51 versus 3.45. We observe similar results when comparing both models on a set of relevant molecular properties such as lipophilicity (log*P*), topological polar surface area (TPSA) and number of aromatic rings and the synthesizability of generated molecules against the test set shown in Extended Data Fig. 2, bottom. Although PILOT shows similar distributions for both log*P* and TPSA, it is substantially worse in reproducing the number of aromatic rings and similarly synthesizable compounds compared to the test set. In Supplementary Fig. 2, we provide additional results comparing FLOWR and PILOT on the SPINDR test set using key drug-likeness filters proposed by ref. ^40^ (inspired by ref. ^41^). These findings demonstrate that ligands generated by PILOT exhibit up to 40% lower pass-through rates compared to those generated by FLOWR, with FLOWR’s results aligning substantially more closely with the SPINDR test data.

In Supplementary Table 3, we repeat the same experiments while incorporating explicit hydrogens in the ligands for both training and inference. Under these conditions, PILOT exhibits a clear decrease in performance, whereas FLOWR maintains comparable results. However, for both models validity drops substantially, with RDKit-validity decreasing to 0.64 ± 0.48 for FLOWR and 0.52 ± 0.50 for PILOT, whereas PB-validity declines to 0.60 ± 0.22 and 0.47 ± 0.14, respectively. Notably, strain energy metrics improve substantially with explicit hydrogen modeling for both models. Although heavy-atom-only approaches require post hoc hydrogen addition potentially leading to artificially inflated strain energies, analysis of individual PoseBusters metrics reveals that reduced validity stems predominantly from ligand–protein clashes rather than internal molecular geometry issues. As SPINDR provides limited coverage of both chemical and conformational space, we anticipate these clashes will diminish with increased training data and, critically, by modeling explicit hydrogens also in protein pockets alongside ligands.

Overall, the proposed FLOWR model consistently outperforms PILOT across all evaluated metrics, demonstrating substantially improved capability in modeling and generalizing ligand–pocket complex distributions. Specifically, we observe an average increase of approximately 15% in ligand and ligand–pocket validity metrics, along with substantially improved AutoDock-Vina scores, indicating higher-quality generated poses. Interestingly, using FLOWR with only 50 inference steps consistently yields better results and yields comparable or slightly worse results with 20 inference steps compared to the PILOT model with 500 steps. Thus, FLOWR achieves substantial performance gains while reducing inference time by up to a factor of 70.

Nevertheless, there remains room for improvement, particularly in reducing strain energies of generated ligands. Moreover, accurately modeling ligand–pocket complexes with explicit hydrogens continues to be challenging, especially in scenarios with limited training data. We encourage the scientific community to evaluate generative models incorporating explicit hydrogen atoms as a challenging benchmark in future research. In this context, the proposed SPINDR dataset represents a valuable resource, providing a robust and comprehensive benchmark for evaluating and comparing ligand generation models.

In SBDD, understanding how a ligand interacts with its target binding site at the atomic level is essential for optimizing potency, selectivity and pharmacological properties^33,42,43^. Ligand–pocket interactions—including hydrogen bonds, hydrophobic contacts, *π*–*π* and *π*–cation stacking, salt bridges and electrostatic or van der Waals interactions—collectively determine binding affinity and specificity. Consequently, these protein–ligand interactions or, more precisely, the ligand’s binding pose are crucial for assessing biological relevance and activity^29^. To systematically identify such interactions, protein–ligand interaction fingerprints (PLIFs) are commonly employed^29,33^. PLIFs encode key interaction features, including the interacting protein residue, interaction type and, optionally, the ligand atom involved^29,33^.

**Extended Data Fig. 4:**
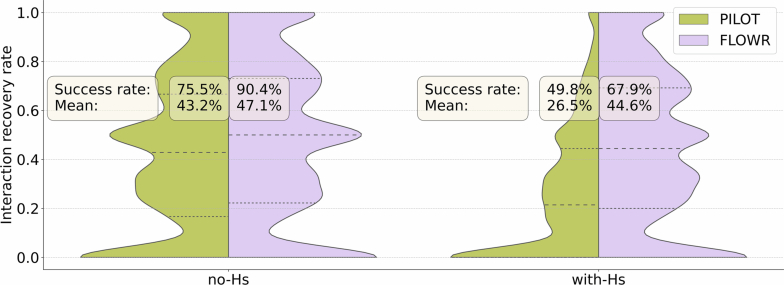
Comparison of PILOT and FLOWR on interaction recovery on SPINDR. Comparison of PILOT and FLOWR in terms of interaction recovery rate distributions across the SPINDR test set targets sampling 100 ligands per target, shown as violin plots with embedded box plots. Both models are either trained without explicit hydrogens (no-Hs) or with explicit hydrogens (with-Hs). The success rate is the percentage of ligands for which interaction fingerprints could be retrieved. In the embedded box plots, the center line represents the median, box bounds represent the 25th and 75th percentiles (interquartile range, IQR), and whiskers extend to 1.5x IQR from the box bounds. Green color denotes PILOT and pink FLOWR distributions. Interaction fingerprints were calculated using ProLIF. Source data

In contrast to previous studies, we closely investigate our proposed model’s capability to reproduce interactions observed in reference ligands. Extended Data Fig. 4 illustrates the distribution of interaction recovery rates for PILOT and FLOWR across the SPINDR test set targets, both with and without explicit hydrogen modeling, using the same sampling settings as before. We also report the success rate, defined as the proportion of RDKit- and PoseBusters-valid ligands for which interactions could be identified. As shown, FLOWR consistently outperforms PILOT (47.1% versus 43.2%, with success rates of 90.4% versus 75.5%), particularly when explicitly modeling hydrogens (42.5% versus 26.5%, with success rates of 67.9% versus 49.8%).

However, these results suggest that a purely de novo generation approach may be less suitable for targeted ligand generation tasks commonly employed in hit expansion and optimization campaigns. To address this, we propose using FLOWR.MULTI (see above), a multi-purpose model capable of interaction-conditional generation. We present detailed results for this approach in the following section.

To improve interaction recovery, we propose to use FLOWR.MULTI with an interaction-based fragmentation for training and inference, which ensures that in inpainting-mode ligand atoms involved in pocket interactions are kept fixed. Let ${{\bf{X}}}_{{\rm{p}}}=\{{{\bf{x}}}_{{\rm{p}},j}\in {{\mathbb{R}}}^{3}:j=1,\ldots ,{n}_{{\rm{p}}}\}$ denote the 3D coordinates of the *n*_p_ pocket atoms, ${{\bf{X}}}_{{\rm{l}}}=\{{{\bf{x}}}_{{\rm{l}},i}\in {{\mathbb{R}}}^{3}:i=1,\ldots ,{n}_{l}\}$ denote the ground-truth (native) 3D coordinates of the *n*_l_ ligand atoms, $I\in {\{0,1\}}^{{n}_{{\rm{p}}}\times {n}_{{\rm{l}}}\times {d}_{I}}$ be an interaction tensor, where the entry *I*_*j*,*i*,*k*_ indicates whether pocket atom *j* and ligand atom *i* participate in an interaction of type *k* (with *d*_*I*_ possible interaction channels). We define a binary mask $M\in {\{0,1\}}^{{n}_{{\rm{l}}}}$ by

3 $$\begin{array}{l}{M}_{i}={{ {\mathbb{I}} }}\left\{\mathop{\sum }\limits_{j=1}^{{n}_{{\rm{p}}}}\mathop{\sum }\limits_{k=1}^{{d}_{I}}{I}_{j,i,k} > 0\right\},\,\,\,\,\,\,\,\end{array}i=1,\ldots ,{n}_{{\rm{l}}},$$

where ${{ {\mathbb{I}} }}\{\cdot \}$ denotes the indicator function. This mask partitions the ligand atoms into a set of interacting atoms (superscript I) for which we fix the flow time to *t*_*I*_ = 1 and set the noise to the ground truth ${\bf{l}}_{0}^{I}={\bf{l}}_{1}^{I}$, and a set of remaining atoms that are generated unconstrained, as described above.

**Extended Data Fig. 5:**
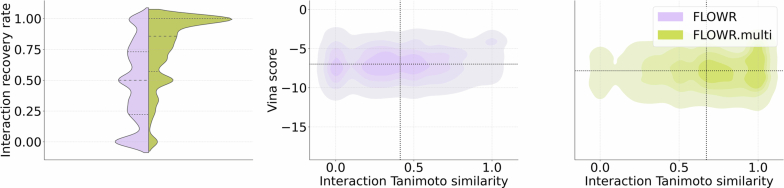
Comparison of FLOWR and FLOWR.multi on interaction similarity and Vina scores on SPINDR. **(Left)** Split violin plot of per-ligand interaction recovery rates across the SPINDR test set targets with 100 sampled ligands per target. Dashed lines indicate the lower quartile (25th percentile), median, and upper quartile (75th percentile); the left half corresponds to FLOWR (pink) and the right half to FLOWR.multi (green). **(Right)** Two-dimensional (2D) kernel density estimates of per-ligand protein–ligand interaction fingerprint (PLIF) Tanimoto similarity versus AutoDock Vina docking score for FLOWR and FLOWR.multi, respectively. Dotted lines indicate the respective mean values. Interaction fingerprints were calculated using ProLIF. Source data

Using FLOWR.MULTI, we achieve an average interaction recovery rate of 76.1%; the distribution compared to the FLOWR model is shown in Extended Data Fig. 5, left. Notably, despite the conditional generation process, the model maintains its ability to explore chemical space, achieving an average molecular diversity of 0.83 compared to 0.86 for FLOWR. As illustrated in Extended Data Fig. 5, right, FLOWR.MULTI also substantially improves predicted binding affinity, as indicated by a lower average Vina score (−7.18 versus −6.93), while interaction Tanimoto similarity nearly doubles.

Thus, we observe that FLOWR.MULTI effectively generates ligands adhering to predefined interaction profiles, improving pose accuracy (as measured by Vina scoring) without substantially compromising chemical diversity or exploration compared to the purely de novo FLOWR model. For a more comprehensive overview of the performance of FLOWR.MULTI on the SPINDR test dataset for interaction-conditional and multi-purpose generation, we refer to Supplementary Information section 2 for detailed results. In the next section, we further investigate the multi-purpose capabilities of FLOWR.MULTI in more details using two test set targets.

To evaluate the multi-purpose generative capabilities of the FLOWR.MULTI model, we randomly selected two targets (PDB ID: 5YEA and 4MPE) from the test set and generated ligands under three distinct conditions: interaction-conditional, scaffold-conditional and functional group-conditional generation. Note that FLOWR.MULTI can also be applied to tasks such as fragment linking and fragment growing; however, for clarity, we leave the evaluation of these additional applications to future work.

The selected crystal structure 5YEA represents lipoprotein-associated phospholipase A2 (Lp-PLA2), a validated therapeutic target implicated in atherosclerosis, Alzheimer’s disease and diabetic macular edema^44^. Potent inhibitors has been found through fragment screening, molecular docking and structure-guided optimization, achieving substantial potency improvements from micromolar to single-digit nanomolar inhibitors^44^. Given the proven effectiveness of structure-based approaches for this target, applying FLOWR.MULTI to Lp-PLA2 (5YEA) is particularly interesting. The other selected protein target, 4MPE, corresponds to pyruvate dehydrogenase kinase (PDK), an enzyme family (isoforms 1–4) that negatively regulates mitochondrial pyruvate dehydrogenase complex activity through phosphorylation^45^. PDK isoforms are clinically relevant, as their overexpression is associated with obesity, diabetes, heart failure and cancer, making them attractive therapeutic targets. Previous works explored a structure-guided approach to design selective inhibitors targeting the conserved ATP-binding pocket of PDK isoforms, resulting in the potent inhibitor PS10^45^, making it an interesting reference point for our FLOWR.MULTI model.

**Extended Data Fig. 6:**
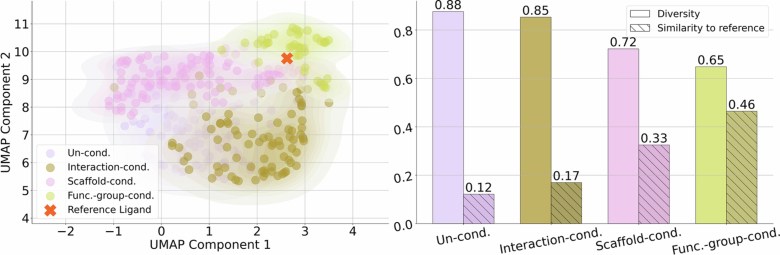
Evaluation of chemical space coverage on 5YEA with FLOWR.multi. Using the unconditional, interaction-, scaffold-, and functional group-conditional generation modes of FLOWR.multi, 100 ligands were sampled for a randomly selected target from the SPINDR test set (here: PDB-ID 5YEA). Chemical space coverage is visualized with respect to the reference ligand using Morgan fingerprints and Uniform Manifold Approximation and Projection (UMAP). Average diversity of the sampled ligand sets and average similarity to the reference are reported. Each generation mode is represented by a distinct color as indicated in the legend. Source data

**Extended Data Fig. 7:**
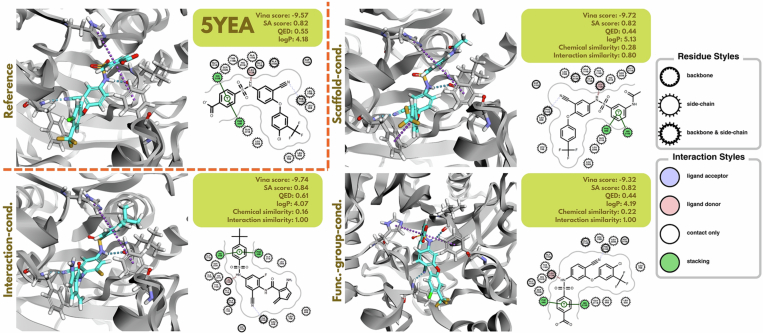
Visualization of conditional generation on 5YEA with FLOWR.multi. Using the interaction-, scaffold-, and functional group-conditional generation modes of FLOWR.multi, 100 ligands for a randomly selected target from the SPINDR test set (here: PDB-ID 5YEA) were sampled. Three ligands were selected at random and compared to the reference compound based on Vina score, synthetic accessibility (SA) score, quantitative estimate of drug-likeness (QED), octanol–water partition coefficient (logP), chemical similarity, and interaction similarity. Atom colors: C (cyan/gray), N (blue), O (red), S (yellow), F (ochre), Cl (green), H (white). Interaction fingerprints were calculated using ProLIF. Interaction diagrams were generated using the OpenEye Python Toolkit. Source data

**Extended Data Fig. 8:**
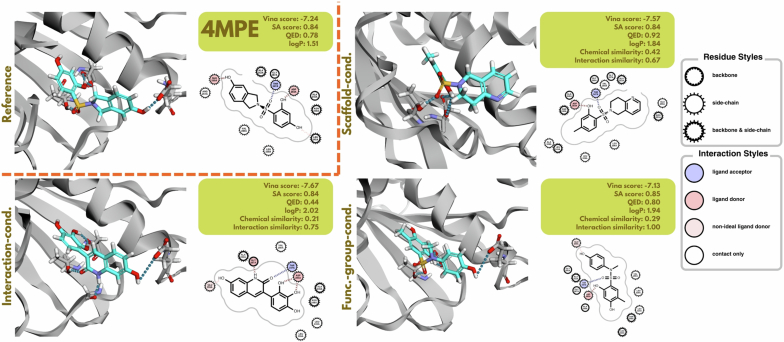
Evaluation of conditional generation on 4MPE with FLOWR.multi. Using the interaction-, scaffold-, and functional group-conditional generation modes of FLOWR.multi, 100 ligands for a randomly selected target from the SPINDR test set (here: PDB-ID 4MPE) were sampled. Three ligands were selected at random and compared to the reference compound based on Vina score, synthetic accessibility (SA) score, quantitative estimate of drug-likeness (QED), octanol–water partition coefficient (logP), chemical similarity, and interaction similarity. Atom colors: C (cyan/gray), N (blue), O (red), S (yellow), F (ochre), Cl (green), H (white). Interaction fingerprints were calculated using ProLIF. Interaction diagrams were generated using the OpenEye Python Toolkit. Source data

For each target and condition, we generated 100 ligands and compared the resulting ligand distributions to the respective reference ligand in terms of PB-validity, Vina docking score, interaction recovery rate and synthetic accessibility. The results are summarized in Supplementary Tables 6 and 7. We consistently observe high PB-validity across targets, suggesting that FLOWR.MULTI effectively learned to generate accurate ligand poses independent of the conditioning mode. Although the mean Vina scores across generated ligands does not match those of the reference ligands, selecting the top-10 ligands based on Vina scores consistently yielded ligands with superior docking scores compared to the references (with slightly worse results for functional group-conditional generation). Interaction recovery rates are generally close to 1, indicating that the generated ligands closely reproduce the interaction profiles of the reference ligands. Finally, the mean SA scores, indicative of synthesizability, are consistently around or above 0.80, comparable to the reference ligands. This suggests that the generated ligands not only satisfy relevant physicochemical criteria but also are likely to be synthetically accessible. In Extended Data Fig. 6, we show the chemical space coverage of generated ligands per generation mode, evaluate the sample diversity and the diversity toward the reference compound. Notably, we find a strong dependence of ligand diversity and reference similarity on the condition-mode. Although in the de novo setting we get the most diverse set of ligands, as to be expected, the interaction-conditional setting also shows a strong chemical space coverage although interaction recovery is substantially enhanced reaching almost 90%. However, especially the functional-group-conditional setting allows for a close resemblance of the reference’s chemical space. This is interesting, as this shows that practitioners can use different conditional setups of FLOWR.MULTI for controlled chemical space exploration. In Extended Data Fig. 7, we visualize a randomly selected ligand for 5YEA per conditioning mode and compare to the reference ligand. Extended Data Fig. 8 shows the corresponding visualization for 4MPE. More examples and visualizations for both targets are provided in Supplementary Information section 2.

## Discussion

Although structure-based generative models have the potential to accelerate early-stage drug discovery by serving as ideation tools from hit exploration to lead optimization, they must be viewed as complementary to—rather than replacements for—medicinal chemistry expertise and experimental validation. The generated molecules represent hypotheses that require rigorous downstream evaluation, including synthesis feasibility assessment, in vitro activity profiling and binding pose confirmation through cocrystallography or cryo-electron microscopy.

Several limitations of the current approach should be acknowledged. First, ligand validity degrades substantially when modeling explicit hydrogens, which we attribute to limited training data coverage and the absence of explicit hydrogen modeling on the protein side. Addressing this will probably require both larger datasets and joint protein–ligand hydrogen modeling during training. Second, although the SPINDR dataset provides high-quality cocrystal structures with careful split design, it offers limited chemical and conformational space coverage compared to the full drug-like molecule landscape; models trained on it may not generalize well to underrepresented chemotypes or binding site topologies. Third, the model operates on static protein structures and does not account for conformational flexibility, induced fit effects or allosteric modulations, which are often critical for accurate binding mode prediction in real drug discovery campaigns. Finally, although strain energies of generated ligands are substantially lower than those of competing approaches, they remain elevated relative to cocrystal reference structures, indicating that further improvements in conformational accuracy are needed.

Several future directions could address these limitations and expand the applicability of flow-based generative models for structure-based drug design. Scaling to larger and more chemically diverse training sets, potentially incorporating data from predicted protein–ligand complexes, could improve coverage of chemical space and enhance explicit hydrogen modeling. Incorporating protein pocket flexibility—for example, by conditioning on ensembles of conformations or integrating molecular dynamics snapshots—would better capture the dynamic nature of protein–ligand interactions. Direct integration of pharmacokinetic property predictors, synthetic accessibility constraints and selectivity objectives into the generative process or as guidance signals during inference would enhance practical utility for medicinal chemistry programs. Prospective experimental validation campaigns, in which generated ligands are synthesized and profiled against their intended targets, are essential to assess real-world performance and identify failure modes not captured by computational benchmarks. In addition, systematic evaluation of FLOWR.MULTI on further fragment-based design tasks, including fragment linking and fragment growing, would provide a more comprehensive assessment of its multi-purpose capabilities.

## Methods

Here, we provide the methodological foundations and notation for the flow matching framework used in FLOWR. Our approach builds on continuous normalizing flows (CNFs) trained via conditional flow matching (CFM)^19,46^, which combines the stable regression objectives of diffusion models with efficient deterministic inference in a simulation-free framework. We specifically employ optimal transport CFM (OT-CFM)^20^ to construct simpler, more stable flows by minimizing transport costs between source and target distributions. Extending this with equivariant flow matching^21^ allows us to exploit the rotational and translational symmetries inherent to molecular systems, yielding flows with shorter integration paths, improved sampling efficiency and natural incorporation of physical symmetries—essential for generating geometrically valid protein–ligand complexes. Below, we detail each of these components and describe how they are integrated into our model.

### Flow matching for continuous data

Flow matching^19,47,48^ is a generative modeling framework based on CNFs. A CNF defines a time-dependent flow ${\phi }_{t}:{{\mathbb{R}}}^{d}\to {{\mathbb{R}}}^{d}$, where *d* is the data dimensionality, through the ordinary differential equation (ODE)

4 $$\frac{d}{dt}{\phi }_{t}(x)={v}_{t}({\phi }_{t}(x)),\,\,\,\,\,\,\,\,\,{\phi }_{0}(x)=x,$$

where ${v}_{t}:{{\mathbb{R}}}^{d}\times [0,1]\to {{\mathbb{R}}}^{d}$ is a time-dependent vector field. The flow *ϕ*_*t*_ pushes forward an initial (prior) distribution *p*_0_ to a target (data) distribution *p*_1_ through the induced time-dependent density path *p*_*t*_. Samples from *p*_1_ are obtained by drawing *x*_0_ ≈ *p*_0_ and integrating the ODE forward from *t* = 0 to *t* = 1.

Training a CNF via maximum likelihood requires simulating the ODE trajectory and computing the divergence of *v*_*t*_, which is computationally expensive. Flow matching provides a simulation-free regression objective by regressing a parameterized vector field ${v}_{t}^{\theta }({x}_{t})$, where *θ* denotes the learnable model parameters, against a target vector field *u*_*t*_(*x*_*t*_)

5 $${{\mathcal{L}}}_{\mathrm{FM}}(\theta )={{\mathbb{E}}}_{t\sim {\mathcal{U}}[0,1],{x}_{t}\sim {p}_{t}({x}_{t})}{||{v}_{t}^{\theta }({x}_{t})-{u}_{t}({x}_{t})||}^{2}.$$

In practice, both the marginal vector field *u*_*t*_(*x*_*t*_) and the marginal probability path *p*_*t*_(*x*_*t*_) are intractable. The crucial insight of CFM^19,20^ is that equivalent gradients can be obtained by conditioning on data samples *x*_1_ ≈ *p*_1_. One defines a conditional probability path *p*_*t*∣1_(*x*_*t*_∣*x*_1_) with an associated conditional vector field *u*_*t*_(*x*_*t*_∣*x*_1_), leading to the CFM objective

6 $${{\mathcal{L}}}_{\mathrm{CFM}}(\theta )={{\mathbb{E}}}_{t\sim {\mathcal{U}}[0,1],{x}_{1}\sim {p}_{1},{x}_{t}\sim {p}_{t| 1}({x}_{t}| {x}_{1})}{||{v}_{t}^{\theta }({x}_{t})-{u}_{t}({x}_{t}| {x}_{1})||}^{2}.$$

A common choice of conditional path is the Gaussian interpolation

7 $${p}_{t| 1}({x}_{t}| {x}_{1})={\mathcal{N}}({x}_{t}| t\,{x}_{1}+(1-t)\,{x}_{0},\,{\sigma }^{2}I\,),$$

where *x*_0_ ≈ *p*_0_ is a sample from the prior distribution, *σ* > 0 is a small noise parameter, and *I* denotes the identity matrix. The corresponding conditional vector field is *u*_*t*_(*x*_*t*_∣*x*_1_) = *x*_1_ − *x*_0_, defining straight conditional paths between noise and data that can be efficiently integrated during sampling.

### Mini-batch optimal transport

The choice of coupling between prior samples *x*_0_ and data samples *x*_1_ substantially affects the geometry of the learned flows. When *x*_0_ and *x*_1_ are coupled independently, that is, (*x*_0_, *x*_1_) ∼ *p*_0_(*x*_0_) *p*_1_(*x*_1_), the resulting marginal vector field can exhibit crossing paths and unnecessarily complex trajectories. OT-CFM^20^ addresses this by replacing the independent coupling with an approximate optimal transport plan *π*(*x*_0_, *x*_1_) that minimizes the expected squared Euclidean transport cost.

Computing the exact OT plan between continuous distributions is intractable in general. OT-CFM therefore approximates the OT coupling at the mini-batch level: given a batch of prior samples $({x}_{0}^{1},\ldots ,{x}_{0}^{B})$ and data samples $({x}_{1}^{1},\ldots ,{x}_{1}^{B})$, one computes the pairwise cost matrix ${M}_{ij}=\parallel {x}_{0}^{i}-{x}_{1}^{\,j}{\parallel }^{2}$ and solves the resulting discrete OT problem to obtain an optimal assignment within the batch. Training pairs are then formed according to this assignment. This mini-batch OT coupling produces straighter conditional paths, more stable training and faster inference since the learned vector fields require fewer integration steps^20^.

### Equivariant optimal transport

For molecular systems that exhibit symmetries under the Euclidean group *E*(3) and the permutation group *S*_*N*_, standard OT-CFM can yield suboptimal transport plans because it does not account for the invariance of the target distribution under rotations, reflections and atom permutations. Equivariant optimal transport flow matching^21^ addresses this by replacing the standard squared Euclidean cost with a symmetry-aware cost function

8 $$\mathop{c}\limits^{ \sim }({x}_{0},{x}_{1})=\mathop{\min }\limits_{g\in G}\parallel {x}_{0}-\rho (\,g)\,{x}_{1}{\parallel }^{2},$$

where *G* is the relevant symmetry group, and *ρ*(*g*) denotes its action on the molecular configuration. For molecules, *G* comprises rotations and reflections *O*(*D*) combined with atom permutations *S*(*N*), where *D* = 3 is the spatial dimension, and *N* is the number of atoms. The cost function aligns each pair of samples along their symmetry orbits before computing the transport cost.

In practice, jointly optimizing over all rotations and permutations is computationally intractable, so the minimization is approximated sequentially^21^: first, the optimal permutation $\widetilde{s}=\arg \,\,{\min }_{s\in {S}_{N}}\parallel {x}_{0}-\rho (s)\,{x}_{1}{\parallel }^{2}$ is found using the Hungarian algorithm; then, the optimal rotation ${R}^{* }=\arg \,\,{\min }_{R\in O(D)}\parallel {x}_{0}-R\,\rho (\widetilde{s})\,{x}_{1}{\parallel }^{2}$ is computed via the Kabsch algorithm. The modified cost matrix is then used within the standard mini-batch OT solver. This equivariant OT procedure produces nearly optimal integration paths even for small batch sizes, leading to shorter paths, reduced integration errors and improved sampling fidelity for molecular systems.

### Discrete flow models

For categorical molecular features such as atom types and bond orders, we adopt the discrete flow model framework^26,49^, which extends flow matching to discrete state spaces via continuous-time Markov chains (CTMCs). Analogously to continuous flow matching, discrete flow model defines a conditional probability path *p*_*t*∣1_(⋅∣*x*_1_) that interpolates between a uniform prior and the data distribution. For a categorical variable *x*_1_ with *K* possible states, the conditional probability at time *t* is given by

9 $${p}_{t| 1}({x}_{t}| {x}_{1})=t\,\delta ({x}_{t},{x}_{1})+(1-t)\,\frac{1}{K},$$

where *δ*(*x*_*t*_, *x*_1_) is the Kronecker delta. At *t* = 0, this reduces to a uniform distribution over all *K* states, whereas at *t* = 1 it concentrates on the data *x*_1_. A neural network learns a data denoiser ${p}_{1| t}^{\theta }(\cdot | {x}_{t})$ that predicts the clean data from the noisy sample, trained by minimizing the cross-entropy between the predicted and true posterior distributions. During inference, the denoiser is used within a CTMC integration scheme that progressively drives *x*_*t*_ from the uniform prior toward the data distribution, analogously to the Euler integration used for continuous variables.

### The SEMLA architecture and FLOWR extensions

The neural network architecture for FLOWR is based on SEMLA^18^, a scalable *E*(3)-equivariant message passing architecture originally proposed in the SEMLAFLOW framework for unconditional 3D molecular generation. SEMLA represents each atom *i* with invariant features ${{\bf{h}}}_{i}\in {{\mathbb{R}}}^{{d}_{\mathrm{inv}}}$ and equivariant features ${{\bf{x}}}_{i}\in {{\mathbb{R}}}^{3\times {d}_{{\rm{equi}}}}$, where *d*_inv_ and *d*_equi_ denote the invariant and equivariant feature dimensions, respectively. Translation invariance is enforced through zero-centering of equivariant features; combined with equivariant updates throughout the network, the learned density is *E*(3)-invariant.

A key innovation in SEMLA is latent attention: invariant node features are projected into a smaller latent space of dimension *d*_*l*_ ≪ *d*_inv_ before computing pairwise messages, reducing the computational complexity of the attention mechanism from ${\mathcal{O}}({N}^{2}{d}_{\,\mathrm{inv}}^{2})$ to ${\mathcal{O}}({N}^{2}{d}_{l}^{2})$, where *N* is the number of atoms. Pairwise messages are computed using a two-layer multilayer perceptron that combines latent invariant features with dot products of equivariant features and are then split into separate invariant and equivariant attention scores. Softmax-normalized attention weights aggregate node features with a variance-preserving scheme.

We extend SEMLA for FLOWR in several ways. First, we incorporate a separate pocket encoder that processes the protein pocket ${\mathcal{P}}$ independently of the flow time *t* and the noisy ligand *l*_*t*_. This encoder uses the same SEMLA layer design and outputs pocket embeddings that are reused across all integration steps during ligand generation, substantially reducing computational cost. Second, we add a cross-attention module within the ligand decoder that takes invariant and equivariant embeddings of ${\mathcal{P}}$, *l*_*t*_ and optionally the interaction matrix ${\mathcal{I}}$, following the same latent attention design for efficiency.

Third, we replace the equivariant feed-forward module in SEMLA with a gated variant. For atom *i* with invariant features ${{\bf{h}}}_{i}\in {{\mathbb{R}}}^{{d}_{{\rm{inv}}}}$ and equivariant features ${{\bf{x}}}_{i}\in {{\mathbb{R}}}^{3\times {d}_{{\rm{equi}}}}$, the gated feed-forward output is

10 $${{\bf{x}}}_{i}^{\,\mathrm{out}}={{\bf{W}}}_{\theta }^{2}{\widehat{{\bf{x}}}}_{i},\,\,\mathrm{where}\,\,{\widehat{{\bf{x}}}}_{i}=\sigma ({\Phi }_{\theta }({{\bf{h}}}_{i},\parallel {{\bf{x}}}_{i}\parallel ))\odot {{\bf{W}}}_{\theta }^{1}{{\bf{x}}}_{i},$$

where *σ* denotes the elementwise sigmoid function, ⊙ is elementwise multiplication, ${{\bf{W}}}_{\theta }^{1},{{\bf{W}}}_{\theta }^{2}\in {{\mathbb{R}}}^{{d}_{\mathrm{equi}}\times {d}_{\mathrm{equi}}}$ are learnable weight matrices and *Φ*_*θ*_ is a two-layer multilayer perceptron mapping invariant features and equivariant norms to gating coefficients. This gated module is substantially faster than the original equivariant feed-forward block. Fourth, we pass bond embeddings into the self-attention module on every layer, as opposed to only the first layer, improving molecular validity with negligible impact on inference time.

We parameterize FLOWR with a 4-layer pocket encoder using ${d}_{\,{\rm{inv}}}^{{\rm{enc}}}=256$ and a 12-layer ligand decoder using ${d}_{\,{\rm{inv}}}^{{\rm{dec}}}=384$. Both encoder and decoder use *d*_equi_ = 64, a latent attention dimension of *d*_*l*_ = 64 and *n*_heads_ = 32 attention heads.

### Training and Inference

We train FLOWR to generate ligands conditioned on a given structure. As 3D molecular graphs contain a mixture of continuous and categorical data types, FLOWR jointly generates continuous and discrete distributions. Our approach follows a similar setup to ref. ^18^. Specifically, we apply the continuous flow matching framework from ref. ^20^ to learn ligand coordinates and the discrete flow models framework from ref. ^26^ to learn atom types and bond orders. Ligand formal charges are not learned through a flow but simply predicted by the model.

### Model training

Training proceeds by sampling ligand noise *l*_0_ ≈ *p*_noise_, a ligand, pocket and interaction tuple $({l}_{1},{\mathcal{P}},{\mathcal{I}})\sim {p}_{\mathrm{data}}$, and a time *t* ∈ [0, 1]. We use Gaussian noise for coordinates and uniform distributions for atom and bond types to create *p*_noise_. Writing *l*_*t*_ = (**x**_*t*_, **a**_*t*_, **b**_*t*_) for the coordinate, atom type and bond order components of the noisy ligand, we sample from the conditional probability path *l*_*t*_ ≈ *p*_*t*∣1_(*l*_*t*_∣*l*_1_) used in ref. ^18^, defined as

11 $$t\sim \,{\mathrm{Beta}}\,(\alpha ,\beta ){{\bf{x}}}_{t}\sim {\mathcal{N}}(t{{\bf{x}}}_{1}+(1-t){{\bf{x}}}_{0},{\sigma }^{2})$$

12 $${{\bf{a}}}_{t}\sim \,\mathrm{Cat}\,\left(t\delta ({{\bf{a}}}_{1})+(1-t)\frac{1}{| A| }\right); {{\bf{b}}}_{t}\sim \,\mathrm{Cat}\,\left(t\delta ({{\bf{b}}}_{1})+(1-t)\frac{1}{| B| }\right).$$

Here Cat(⋅) denotes the categorical distribution, *A* and *B* are the sets of possible values for atom types and bond orders, respectively, and *δ*(⋅) is the one-hot encoding operation applied to each item in a sequence individually. We use values *α* = 2.0, *β* = 1.0 and *σ* = 0.2 for all FLOWR models.

Following refs. ^11,12,17^ we train FLOWR to predict *l*_1_ directly by learning the distribution ${p}_{1| t}^{\theta }({l}_{1}| {l}_{t},{\mathcal{P}},{\mathcal{I}})$. This leads to the same loss function as SEMLAFLOW^18^—we apply a mean-squared error loss for ligand coordinates and cross-entropy losses for atom types, bond orders and formal charges. When the model is conditioned on both ${\mathcal{P}}$ and ${\mathcal{I}}$, the interaction features are provided as additional input to the cross-attention module, enabling the model to attend to both structural pocket information and desired interaction patterns simultaneously. Further implementation details and ablation studies are provided in Supplementary Information section 2.

In addition, during training we apply self-conditioning^50^ to improve generation quality. In self-conditioned training, half of the training batches are processed normally, whereas the other half first generate a preliminary prediction of *l*_1_ from the model, which is then detached from the computation graph and provided as additional conditioning input in the subsequent forward pass. For atom and bond types, the conditioning inputs are softmax-normalized probability distributions over the predicted categorical types.

We also apply equivariant optimal transport^21^ during training to reduce the transport cost between *p*_noise_ and *p*_data_. For each training pair (*l*_0_, *l*_1_), the coordinate noise **x**_0_ is transformed via ${\widehat{{\bf{x}}}}_{0}={f}_{\pi }({{\bf{x}}}_{0},{{\bf{x}}}_{1})$, where *f*_*π*_ applies the permutation and rotation that minimize the squared error between **x**_0_ and **x**_1_, as described in the ‘Equivariant optimal transport’ section above. This alignment reduces transport costs and yields straighter, more efficient integration paths during sampling.

### Fragment extraction

We extract molecular scaffolds using RDKit’s GetScaffoldForMol from the MurckoScaffold implementation, defining functional groups as all atoms not part of the scaffold and linkers as nonring scaffold atoms. To enable diverse fragment-based learning, we additionally employ RDKit’s matched molecular pairs analysis via FragmentMol to decompose molecules into chemically meaningful fragments, randomly sampling from these at each training batch. This strategy allows the model to learn scaffold hopping and fragment growing patterns naturally from the data. For interaction-conditional training, we leverage ProLIF-derived interaction fingerprints precalculated for all complexes in the SPINDR dataset.

### Ligand generation

Given a protein pocket ${\mathcal{P}}$ and, optionally, a desired interaction matrix ${\mathcal{I}}$, we can generate samples from the learned data distribution by setting *l*_*t*_ ← *l*_0_, where *l*_0_ ≈ *p*_noise_ and pushing *l*_*t*_ toward the data distribution by following the learned vector field. Specifically, for molecular coordinates **x**_*t*_ we follow the vector field ${v}_{t}^{\theta }({{\bf{x}}}_{t})=\frac{1}{1-t}({\widetilde{{\bf{x}}}}_{1}-{{\bf{x}}}_{t})$, where ${\widetilde{{\bf{x}}}}_{1}$ is the coordinate component of ${\widetilde{l}}_{1}\sim {p}_{1| t}^{\theta }({l}_{1}| {l}_{t},{\mathcal{P}},{\mathcal{I}})$. We then integrate the vector field using an Euler solver with step size Δ*t* as follows: ${\widetilde{{\bf{x}}}}_{t+\Delta t}={{\bf{x}}}_{t}+\Delta t{v}_{t}^{\theta }({{\bf{x}}}_{t})$. For discrete atom and bond types, integration proceeds analogously: at each step, the model predicts the posterior distribution ${p}_{1| t}^{\theta }({x}_{1}| {x}_{t})$ over categorical types, and *x*_*t*_ is updated toward the data distribution according to the CTMC integration scheme, where the transition rates are derived from the predicted posteriors and the conditional probability path defined in the ‘Discrete flow models’ section.

#### Evaluation

To maintain consistency across models, we used identical random seeds for training, inference and data loading. Moreover, we applied the same sampling and evaluation scripts across all models. For each of the 225 test set targets, we generated 100 ligand samples using a standardized size sampling approach. Specifically, we determined native ligand sizes and applied a uniform sampling scheme, allowing for a size deviation of −25% to +10%. This procedure was performed using the same seed across all models to ensure direct comparability.

### Reporting summary

Further information on research design is available in the Nature Portfolio Reporting Summary linked to this article.

## Supplementary information

Supplementary Information Supplementary Figs. 1–12 and Tables 1–7.

Reporting Summary

Peer Review File

## Source data

Source Data Extended Data Fig. 1 Source data.

Source Data Extended Data Fig. 2 Source data.

Source Data Extended Data Fig. 3 Source data.

Source Data Extended Data Fig. 4 Source data.

Source Data Extended Data Fig. 5 Source data.

Source Data Extended Data Fig. 6 Source data.

Source Data Extended Data Fig. 7 Source data.

Source Data Extended Data Fig. 8 Source data.

Source Data Extended Data Fig. 9 Source data.

Source Data Extended Data Fig. 10 Source data.

## Data Availability

The CrossDocked2020 dataset^24^ with precomputed data splits can be downloaded via GitHub at https://github.com/pengxingang/Pocket2Mol/tree/main/data. The SPINDR dataset can be downloaded via Zenodo at https://zenodo.org/records/15257565 (ref. ^51^). Source data for Extended Data Figs. 1–8 is available with this manuscript. The protein-ligand complexes used as case studies were obtained from the Protein Data Bank under accession codes 5YEA and 4EMP. Source data are provided with this paper.

## Extended data

**Extended Data Table 1:** Evaluation and comparison of FLOWR on CrossDocked2020

Model	PB-valid*↑*	Strain*↓*	Vina score*↓*	${{\bf{Vinascore}}}^{{\bf{m}}{\bf{i}}{\bf{n}}}$ *↓*	BondA.W1*↓*	BondL.W1 [10^−2^]*↓*	Size	Time (s)*↓*
POCKET2MOL	0.76 ± 0.39	147.22 ± 61.41	-4.72 ± 1.47	-5.80 ± 1.26	2.04	0.66	17.04 ± 4.11	2320 ± 45
DIFFSBDD	0.38 ± 0.46	519.03 ± 251.32	-2.97 ± 5.21	-4.71 ± 3.30	7.00	0.51	24.85 ± 8.94	160.31 ± 73.30
TARGETDIFF	0.57 ± 0.46	294.89 ± 136.32	-5.20 ± 1.79	-5.82 ± 1.60	7.76	0.42	22.79 ± 9.46	3228 ± 121
DRUGFLOW	0.75 ± 0.39	120.21 ± 73.28	-5.66 ± 1.78	-6.10 ± 1.62	2.11	0.38	21.14 ± 6.81	-
PILOT	0.83 ± 0.33	110.48 ± 87.47	-5.73 ± 1.72	-6.21 ± 1.65	1.75	0.33	22.58 ± 9.77	295.42 ± 117.35
FLOWR	0.92 ± 0.22	87.83 ± 74.30	-6.29 ± 1.56	-6.48 ± 1.45	0.96	0.27	22.28 ± 9.78	12.05 ± 8.01
Test set	0.95 ± 0.21	75.62 ± 57.29	-6.44 ± 2.74	-6.46 ± 2.61	-	-	22.75 ± 9.90	-

Benchmark comparison of the proposed FLOWR model against Pocket2Mol, TargetDiff, DiffSBDD, PILOT and DrugFlow on the CrossDocked2020 test dataset. We follow the conventions in this field and sample 100 ligands per test target, of which there are 100. We evaluate the most expressive metrics, namely PoseBusters-validity, GenBench3D strain energy, AutoDock-Vina scores and the Wasserstein distance of the generated ligands’ bond angles (BondA.W1) and bond lengths (BondL.W1) distributions relative to the test set. For all values, we report the mean across ligands and targets and the average standard deviation across targets as subscripts. For all models, we ran all evaluations on the subset of RDKit-valid ligands. Arrows (up, down) indicate that higher or lower values are preferred, respectively.

**Extended Data Table 2:** Evaluation and comparison of PILOT and FLOWR on SPINDR

Model	Strain energy *↓*	Vina score *↓*	${{\bf{Vinascore}}}^{{\bf{m}}{\bf{i}}{\bf{n}}}$ *↓*	BondA.W1 *↓*	BondL.W1 [10^−2^] *↓*	DihedralW1 *↓*
PILOT	120.10 ± 71.61	-6.30 ± 0.96	-6.68 ± 1.07	1.82	0.42	5.52
FLOWR^20 steps^	134.70 ± 77.58	-6.61 ± 0.98	-6.92 ± 0.96	1.55	0.74	4.67
FLOWR^50 steps^	98.47 ± 56.64	-6.83 ± 0.93	-7.13 ± 0.93	1.18	0.51	4.04
FLOWR^100 steps^	90.05 ± 52.18	-6.93 ± 0.92	-7.22 ± 0.92	1.08	0.35	3.88
Test set	43.27 ± 41.85	-7.69 ± 2.00	-7.88 ± 2.00	-	-	-

Benchmark comparison of the proposed FLOWR model against the PILOT model using the SPINDR test dataset, which consists of 225 targets. For FLOWR results are reported for inference steps of 20, 50, and 100. For both models, 100 ligands were sampled per target. The evaluation includes strain energy calculated with GenBench3D and AutoDock-Vina scores (kcal/mol). For all values, we report the mean across ligands and targets and the average standard deviation across targets as subscripts. Additionally, we report the Wasserstein distance of the generated ligands’ bond angles (BondA.W1), bond lengths (BondL.W1) and dihedral angles (DihedralW1) distributions relative to those in the SPINDR test set. Ligand sizes for all models are sampled uniformly with a -10%/+10% margin around the respective reference ligand size. Arrows (up, down) indicate that higher or lower values are preferred, respectively.

## Code availability

The source code used in this study is available under the MIT License via GitHub at https://github.com/jule-c/flowr and via Zenodo at 10.5281/zenodo.18668261 (ref. ^52^). Model weights can be downloaded via Zenodo at https://zenodo.org/records/15257565 (ref. ^53^).
