# Supplementary material for: FLOWR: flow matching for structure-aware de novo, interaction- and fragment-based ligand generation
Source: Nat Comput Sci. 2026 May 28;6(6):565–74. doi: 10.1038/s43588-026-00998-8 (PMC13293864; doi:10.1038/s43588-026-00998-8)
Supplement: Supplementary file 1 — Supplementary Figs. 1–12 and Tables 1–7. [file 43588_2026_998_MOESM1_ESM.pdf]

nature computational science

Supplementary information https://doi.org/10.1038/s43588-026-00998-8

FLOWR: flow matching for structure-aware de novo, interaction- and fragment-based ligand generation

In the format provided by the authors and unedited

Contents

Additional Details on the SPINDR Dataset . . . 2

1.1 SPINDR VS. CROSSDOCKED . . . 2

Additional Experimental Results . . . 3

2.1 FLOWR VS. PILOT . . . 4

2.2 Performance of FLOWR.MULTI on SPINDR . . . 5

2.3 Strain Analysis . . . 5

2.4 Interactions . . . 6

2.5 Interactions Per Target . . . 7

2.6 4MPE: Visualizations . . . 9

2.7 5YEA: Visualizations . . . 10

Supplementary Section 1: Additional Details on the SPINDR Dataset

1.1 SPINDR VS. CROSSDOCKED

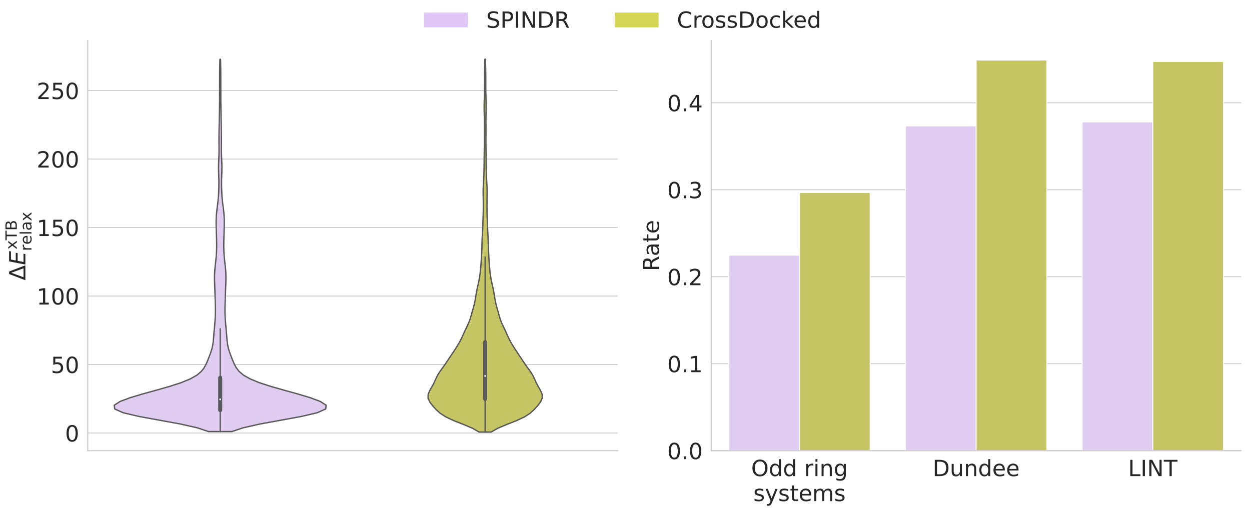

Supplementary Figure 1: Comparison of the SPINDR and CROSSDOCKED2020 datasets in terms of GFN2-xTB relaxation energies computed using the ALPB solvation model (left), as well as the proportion of unusual ring systems and potentially reactive functional groups identified according to two distinct substructure libraries, Dundee and LINT.

Supplementary Figure 1 compares the quality of ligands in the SPINDR dataset against those in the CROSSDOCKED2020 dataset, using three distinct metrics: relaxation energies using GFN2-xTB [2] together with the ALPB solvation model [4], frequency of odd ring systems, and presence of potentially reactive functional groups [7]. Lower delta relaxation energies indicate higher conformational quality as ligands require less structural rearrangement upon binding. As illustrated, the SPINDR dataset exhibits substantially lower delta relaxation energies compared to CROSSDOCKED2020. Additionally, following [7], odd ring systems are defined as ring structures that occur infrequently (fewer than 100 occurrences) within the ChEMBL database, indicating unusual or potentially problematic chemical motifs. Reactive functional groups were identified using standard medicinal chemistry filters, specifically the Dundee and LINT substructure collections. On all three metrics, SPINDR shows on average better values than the CROSSDOCKED2020 dataset.

Supplementary Table 1 further compares the ligand diversity of SPINDR to the standard 100K CROSSDOCKED benchmark dataset. While SPINDR contains fewer systems than CROSSDOCKED, it actually contains more unique ligands, and substantially more unique ligand scaffolds. Ultimately, this is due to CROSSDOCKED being a synthetically-generated dataset created by cross-docking systems from PDBBind, which contains approximately 19K systems in total.

Supplementary Table 1: Ligand comparison in benchmark datasets. Scaffolds were obtained using RDKit's Murcko Scaffold utilities.

Metric	CROSSDOCKED2020	SPINDR
Total ligands	100,563	35,666
Unique ligands	8,433	11,689
Unique ligand scaffolds	4,855	7,713

Supplementary Table 2: Sizes of train, validation and test dataset splits for the three proposed versions of the SPINDR dataset.

Dataset	Train Systems	Val Systems	Test Systems
SPINDR	35,373	68	225
SPINDR^{RMSD}	24,885	68	225
SPINDR$^{\text{RMSD-SEQID}}$	20,349	68	225

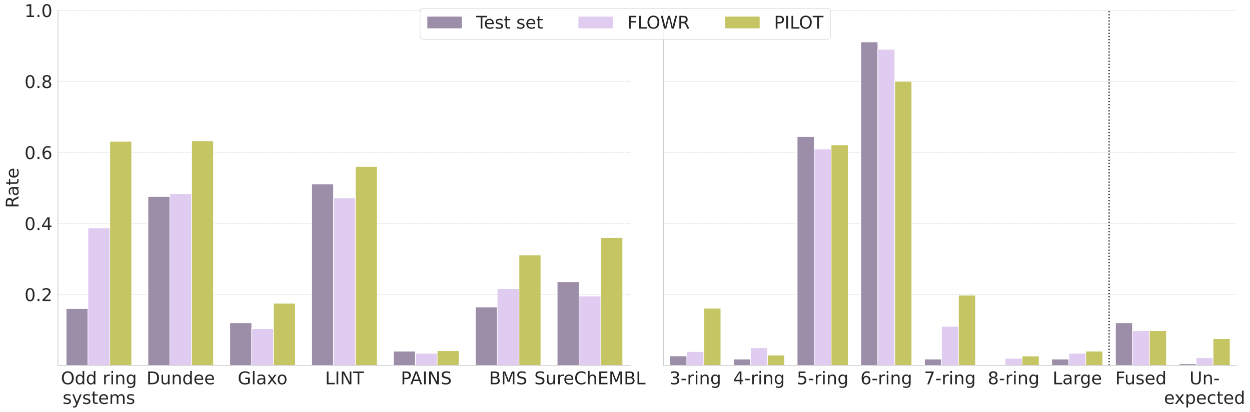

Supplementary Figure 2: **Comparison of FLOWR and PILOT using the 'Walters'-filter and ring distribution analysis.** We assess the distribution learning capabilities of FLOWR and compare its performance against PILOT, utilizing established medicinal chemistry filters provided by the USEFUL_RDKIT_UTILS toolkit (left panel). Specifically, we employed the REOS filter [7] and the RINGSYSTEMLOOKUP to evaluate the generated ligands. Additionally, we present a comparative analysis of ring distributions (right panel). All reported metrics represent mean values computed from 100 ligands sampled per target using the SPINDR test dataset, which comprises 225 distinct test set targets. Ligand sizes were sampled uniformly around the ground truth ligand size, allowing for deviations within a range of ±10%, using a consistent random seed across models.

Like existing datasets of protein-ligand complexes, the SPINDR training set contains many redundant systems – systems which have substantial structural similarity to another training system. Understanding the impact of this redundancy on model performance is a relatively unexplored topic but could have an important influence on the design of future datasets. We therefore apply two data deduplication strategies to SPINDR and report results on all three datasets. Deduplication is only applied to the training data and all models are evaluated identically.

Our initial deduplication strategy works by creating groups of systems such that all systems within the group have identical ligands (based on their canonical SMILES after hydrogen atoms have been removed) and identical pockets atoms where the pocket coordinates are within an RMSD of 1.0 of some reference system for the group. We find that for system groups defined like this the distribution of RMSD values to the reference is very close to zero, so the choice of reference system and the RMSD threshold is not so important. In practice we iterate over all systems in the dataset, if a system cannot be added to an existing group a new group is created with this system as the group's reference system. Once all systems in the training dataset have been grouped a single system is randomly selected from each group to form the deduplicated training set. We refer to this dataset as $\text{SPINDR}^{\text{RMSD}}$. We also explore an extension of this deduplication strategy which allows systems to be in the same group if the sequence identity between a query pocket and the pocket of the reference system for a group is greater than 90%. In this case the RMSD between the query and reference pockets is taken by comparing the coordinates only on matching residues. Again, once groups have been constructed, a single system is randomly sampled from each group to form the deduplicated training set. We refer to this dataset as $\text{SPINDR}^{\text{RMSD-SEQID}}$. The sizes of the three versions of the dataset are shown in Supplementary Table 2.

Supplementary Section 2: Additional Experimental Results

Benchmarking newly proposed models and architectures in the context of structure-based drug design requires careful consideration of multiple evaluation aspects. In addition to the results presented in the main text, we provide a broader assessment using various metrics and evaluation settings in the following sections. Specifically, we evaluate the novelty of generated ligands with respect to the training set, as well as the average uniqueness and diversity among the 100 generated ligands per target. To ensure a comprehensive analysis, we consider both SMILES string- and ECFP4-based measures for uniqueness and diversity. Additionally, following [1], we extend this analysis to include conformer-based uniqueness and diversity. As indicators of drug-likeness, we report RDKit's Quantitative Estimate of Drug-likeness (QED), the Synthetic Accessibility Score (SAScore) [6], molecular weight, logP values, and compliance with Lipinski's Rule of Five.

Supplementary Table 3: Benchmark of the proposed FLOWR model against the recent state-of-the-art diffusion-based PILOT model on the SPINDR dataset. We report RDKit- and PoseBusters-validity of generated ligands, the GenBench3D strain energy and the AutoDock-Vina score. We also state the Wasserstein distance of generated ligands for the bond angles and bond lengths distribution to the SPINDR test set. Novelty, uniqueness and diversity measure the capability of the model to explore the chemical space both in 2D and 3D. RDKit's QED evaluation, SAScore, the molecular weight as well as the logP values evaluate drug-likeness of generated ligands. All presented values are mean values taken for 100 sampled ligands per test set target. The test dataset comprises 225 test set targets. Ligand sizes were drawn from a uniform distribution around the ground truth ligand size allowing for a deviation of -10% and + 10% with the same random seed for all models. Note, both RDKit- and PoseBusters-validity are evaluated on the raw generated set of 100 ligands per target. All other metrics are calculated on the subset of RDKit-valid ligands.

METRIC	TEST SET	PILOT$^{\text{NO-Hs}}$	PILOT$^{\text{WITH-Hs}}$	FLOWR$^{\text{NO-Hs}}$	FLOWR$^{\text{WITH-Hs}}$
RDKIT-VALIDITY	1.00 ± 0.00	0.79 ± 0.39	0.52 ± 0.50	0.94 ± 0.24	0.64 ± 0.48
PB-VALIDITY	0.99 ± 0.02	0.71 ± 0.18	0.47 ± 0.14	0.88 ± 0.21	0.60 ± 0.22
STRAIN ENERGY	43.27 ± 41.85	120.10 ± 71.61	53.07 ± 22.84	90.05 ± 52.18	54.11 ± 33.36
VINA SCORE	-7.69 ± 2.00	-6.30 ± 0.96	-5.00 ± 0.65	-6.93 ± 0.92	-6.48 ± 0.87
VINA SCORE (MINIMIZED)	-7.88 ± 2.00	-6.68 ± 1.07	-5.50 ± 0.66	-7.22 ± 0.92	-6.86 ± 0.87
BONDANGLESW1	-	1.82	2.81	1.08	0.82
BONDLENGTHSW1 [10^{-2}]	-	0.42	0.10	0.35	0.11
NOVELTY	1.00 ± 0.00	0.99 ± 0.10	1.00 ± 0.00	0.94 ± 0.23	1.00 ± 0.00
UNIQUENESS2D	0.92 ± 0.10	0.99 ± 0.05	1.00 ± 0.02	0.94 ± 0.13	0.97 ± 0.07
UNIQUENESS3D	-	0.66 ± 0.20	0.59 ± 0.19	0.50 ± 0.20	0.55 ± 0.17
DIVERSITY2D	0.92 ± 0.04	0.89 ± 0.03	0.90 ± 0.02	0.86 ± 0.05	0.87 ± 0.06
DIVERSITY3D	-	0.25 ± 0.13	0.13 ± 0.19	0.21 ± 0.12	0.18 ± 0.11
SA	0.66 ± 0.12	0.63 ± 0.12	0.64 ± 0.10	0.67 ± 0.13	0.65 ± 0.10
QED	0.49 ± 0.22	0.51 ± 0.21	0.53 ± 0.18	0.52 ± 0.21	0.53 ± 0.21
RINGS	2.98 ± 1.42	2.52 ± 1.42	1.52 ± 0.98	2.68 ± 1.35	2.64 ± 1.43
AROMATIC RINGS	1.84 ± 1.31	1.12 ± 1.07	1.21 ± 0.95	1.52 ± 1.16	1.59 ± 1.22
HACCEPTORS	7.30 ± 4.49	6.19 ± 3.30	5.46 ± 2.21	6.67 ± 4.23	6.47 ± 3.64
HDONORS	2.62 ± 1.68	2.52 ± 1.65	1.55 ± 1.27	2.52 ± 1.68	2.66 ± 1.58
LOGP	0.29 ± 3.48	0.45 ± 3.08	-0.03 ± 2.33	0.29 ± 3.31	0.34 ± 2.99
MOLWT	390.43 ± 119.82	336.79 ± 107.86	337.30 ± 83.59	350.10 ± 114.00	336.09 ± 108.60
LIPINSKI	4.00 ± 1.34	4.45 ± 0.93	4.73 ± 0.55	4.35 ± 1.05	4.32 ± 1.05

2.1 FLOWR VS. PILOT

In Supplementary Table 3, we report the results comparing PILOT and FLOWR for both settings, without explicit and with explicit hydrogens in training and inference, respectively. On average, PILOT shows higher novelty, uniqueness and diversity values of generated ligands. However, in light of the substantially worse results across distribution and ligand-pocket-centric metrics, it is likely that PILOT has a stronger tendency to hallucinate and thus generates physically less plausible, but more diverse structures with higher strains. Regarding RDKit-based ligand property metrics, both models show similar results, while FLOWR shows in general a higher overlap with the test set values indicating better distribution learning capabilities.

Additionally, we compare FLOWR and PILOT in terms of drug-likeness filtering using the 'Walters'-filter [7], which evaluates generated compounds for unusual ring systems (by comparing their frequencies against ring systems found in ChEMBL) and identifies problematic functional groups through substructure matching against established filter collections, including Dundee, Glaxo, LINT, PAINS, BMS, and SureChEMBL. Supplementary Figure 2 (left) illustrates the performance comparison between FLOWR and PILOT based on these drug-likeness criteria. We observe that FLOWR consistently outperforms PILOT, in some cases by substantial margins, and generates compounds whose properties align more closely with those observed in the test set. Furthermore, Supplementary Figure 2 (right) presents a comparative analysis of ring distributions, demonstrating that FLOWR again achieves substantially better overlap with the SPINDR test set distribution compared to PILOT.

Supplementary Table 4: **Evaluation and comparison of PILOT and FLOWR on SPINDR with explicit hydrogens.** Benchmark comparison of the proposed FLOWR model against the PILOT model on the SPINDR test dataset with explicit hydrogens in training and inference. For both models, 100 ligands were sampled per target and evaluated in terms of strain energy, AutoDock-Vina scores (kcal/mol) and Wasserstein distances of generated ligands' bond angle and bond length distributions relative to those in the test set.

MODEL	STRAIN ENERGY ↓	VINA SCORE↓	VINA SCORE^{MIN} ↓	BONDANGLESW1 ↓	BONDLENGTHSW1 [10^{-2}] ↓
PILOT	53.07 ± 22.84	-5.00 ± 0.65	-5.50 ± 0.66	2.81	0.2
FLOWR$^{100\ \text{STEPS}}$	54.11 ± 33.36	-6.48 ± 0.87	-6.86 ± 0.87	0.82	0.1
TEST SET	43.27 ± 41.85	-7.69 ± 2.00	-7.88 ± 2.00	-	-

Supplementary Table 5: We evaluate FLOWR.MULTI for different conditional modes, namely for interaction-, scaffold-, functional-group- and linker-conditional generation, on the SPINDR test set. We report RDKit- and PoseBusters-validity of generated ligands, the GenBench3D strain energy and the AutoDock-Vina score. We also state the pocket-ligand interaction fingerprint recovery rate and Tanimoto similarity using PROLIF, and the Wasserstein distance of generated ligands for the bond angles and bond lengths distribution to the SPINDR test set. Novelty, uniqueness and diversity measure the capability of the model to explore the chemical space both in 2D and 3D. RDKit's QED evaluation, SAScore, the molecular weight as well as the logP values evaluate drug-likeness of generated ligands. All presented values are mean values taken for 100 sampled ligands per test set target. The test dataset comprises 225 test set targets. Ligand sizes were taken from the respective reference ligand and are not sampled. Note, both RDKit- and PoseBusters-validity values are evaluated on the generated set of 100 ligands per target. All other metrics are calculated on the subset of RDKit-valid ligands.

METRIC	FLOWR.MULTI$^{\text{interact.-cond.}}$	FLOWR.MULTI$^{\text{scaffold-cond.}}$	FLOWR.MULTI$^{\text{f.-group-cond.}}$	FLOWR.MULTI$^{\text{linker-cond.}}$
RDKIT-VALIDITY	0.93 ±0.25	0.93 ±0.25	0.92 ±0.26	0.92 ±0.25
PB-VALIDITY	0.86 ±0.19	0.88 ±0.13	0.86 ±0.17	0.85 ±0.17
VINA SCORE	-7.18 ±0.83	-7.41 ±0.67	-7.10 ±0.71	-7.35 ±0.56
VINA SCORE (MINIMIZED)	-7.48 ±0.80	-7.72 ±0.59	-7.34 ±0.72	-7.57 ±0.56
STRAIN ENERGY	107.60 ±93.07	86.26 ±78.31	105.32 ±95.47	94.56 ±84.08
PLIF RECOVERY RATE	0.75 ±0.08	0.65 ±0.11	0.79 ±0.12	0.79 ±0.08
PLIF TANIMOTO SIMILARITY	0.66 ±0.09	0.62 ±0.10	0.74 ±0.13	0.76 ±0.09
BONDANGLESW1	1.17	0.84	1.14	0.91
BONDLENGTHSW1 [10^{-2}]	0.43	0.52	0.58	0.69
NOVELTY	0.93 ±0.26	0.94 ±0.23	0.87 ±0.33	0.87 ±0.33
UNIQUENESS2D	0.83 ±0.26	0.74 ±0.28	0.70 ±0.33	0.53 ±0.31
UNIQUENESS3D	0.40 ±0.21	0.35 ±0.12	0.31 ±0.20	0.26 ±0.17
DIVERSITY2D	0.82 ±0.08	0.77 ±0.07	0.78 ±0.08	0.75 ±0.06
DIVERSITY3D	0.06 ±0.07	0.02 ±0.01	0.07 ±0.12	0.03 ±0.05

2.2 Performance of FLOWR.MULTI on SPINDR

Here we report the evaluation results for FLOWR.MULTI using different conditional generation settings on the SPINDR test dataset. Supplementary Table 5 shows an overview of a set of evaluation metrics to assess pose quality and distribution learning capabilities.

2.3 Strain Analysis

To assess the physical realism of generated conformations, we performed energy minimization on all predicted poses. The optimization substantially reduced molecular strain energy, corresponding to a mean reduction of 62.75 kcal/mol. Importantly, this strain relief was achieved with minimal structural perturbation, as evidenced by the low RMSD of 0.78 ± 0.14 Å between pre- and post-minimization conformations (see Supplementary Figure 3). The refined poses exhibited improved molecular quality metrics, with PoseBusters validity increasing to 0.95 ± 0.08 and Vina score improving to -6.97 ± 0.89 kcal/mol. These results demonstrate that while the model generates physically plausible binding modes, local energy minimization can effectively relieve residual strain without fundamentally altering the predicted protein-ligand interactions.

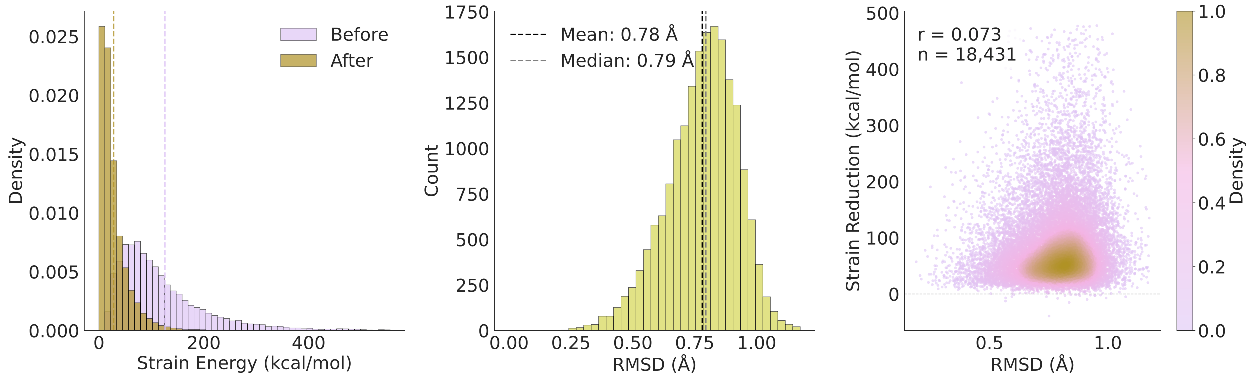

Supplementary Figure 3: **Strain analysis on the SPINDR test set.** We run MMFF94s-based relaxation using RDKit on all generated ligands across the SPINDR test set targets with protein pockets being fixed, and show pre- and post-relaxation strain energies and RMSDs.

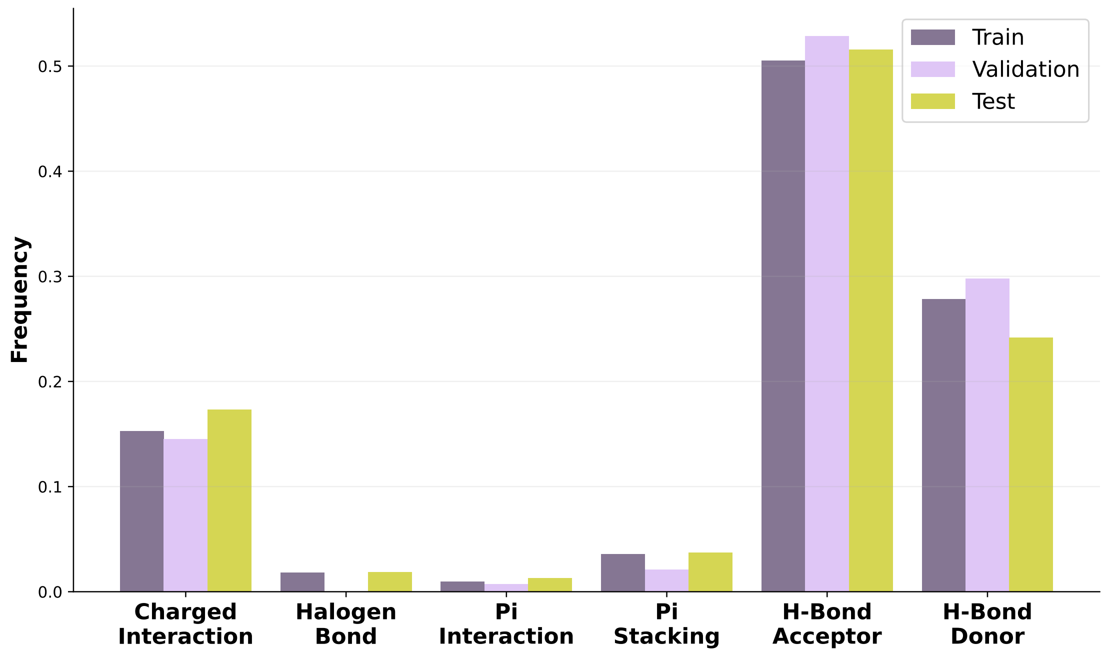

Supplementary Figure 4: Distribution of interaction types on the train, validation and test sets of the SPINDR data that we considered in this work. Charged Interactions refers to either anionic or cationic interactions, and Pi Interactions refer to either cation-pi or pi-cation interactions. The SPINDR dataset contains all possible interactions supported by ProLIF, but we focus on the above interactions for conditional generation since they are the most applicable for small molecule binding.

2.4 Interactions

Following [5], we consider a subset of interaction types in this work extracted using ProLIF [3], including H-bonds (ligand acceptor and ligand donor), π-π stacking, halogen bonds (ligand donor), π-cation (ligand π / protein +), cation-π (ligand + / protein π), anionic (ligand - / protein +), and cationic (ligand + / protein -) interactions. The distribution of these interactions within the SPINDR dataset is shown in Supplementary Figure 4. Notably, interaction sparsity is high, with an average of 99.85% of ligand-protein atom pairs exhibiting no interactions.

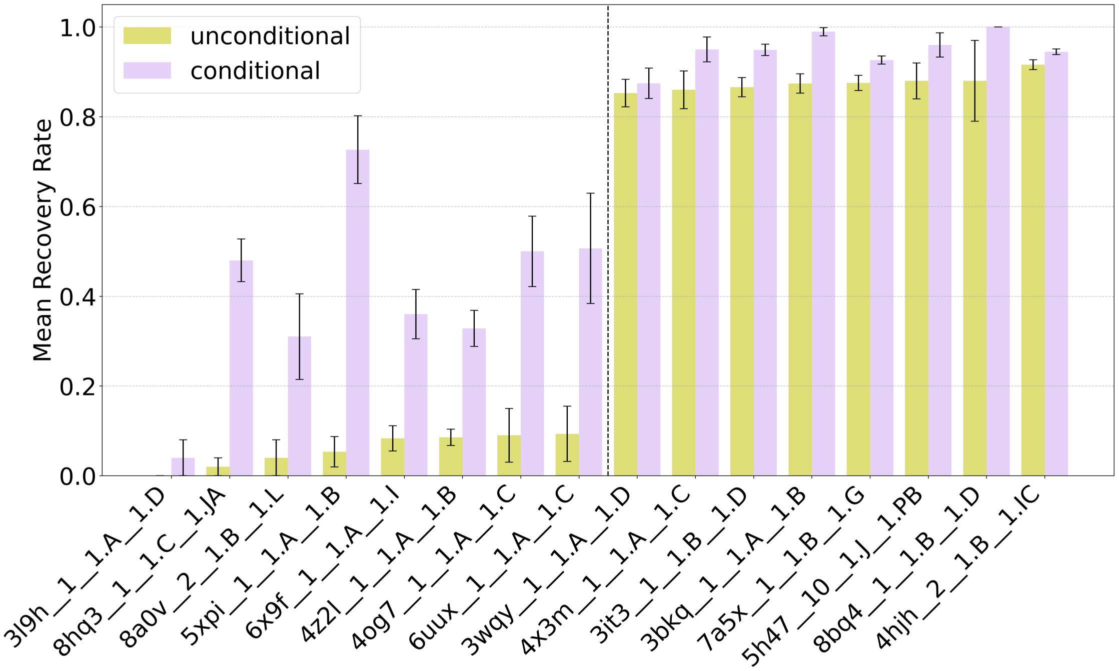

Supplementary Figure 5: Comparison between Flowr and Flowr.multi. We identify eight targets with the lowest (left) and highest (right) average interaction recovery rates under the unconditional Flowr model. For these selected targets, we compare the performance of the Flowr.multi model to assess the impact of conditioning on pocket-ligand interactions.

2.5 Interactions Per Target

To better evaluate the effectiveness of the proposed interaction-conditional training and sampling, we compare Flowr with Flowr.multi models on a per-target basis. Given that the test set comprises 225 targets, visualizing results for all targets is impractical. Instead, we select eight targets with the lowest and with the highest mean interaction recovery rates, as determined by the unconditional model, and compare the corresponding results obtained using the conditional model. This comparison is presented in Supplementary Figure 5. Notably, the conditional model consistently improves interaction recovery across targets where the unconditional model struggled to generate ligands with meaningful interactions. Additionally, it achieves substantially better results even for the top-performing targets, demonstrating that interaction-conditional generation effectively enhances ligand design with pre-specified interaction patterns.

Supplementary Figure 6 presents an example of interaction profiling using the reference ligand of protein 6UUX alongside three randomly selected ligands generated by the interaction-conditional mode of Flowr.multi model. The reference ligand forms two cationic interactions and one H-bond (ligand donor) interaction with ASP149, as well as two H-bond (ligand donor) interactions with ASP93. Notably, all of these interactions are successfully recovered in the generated ligands.

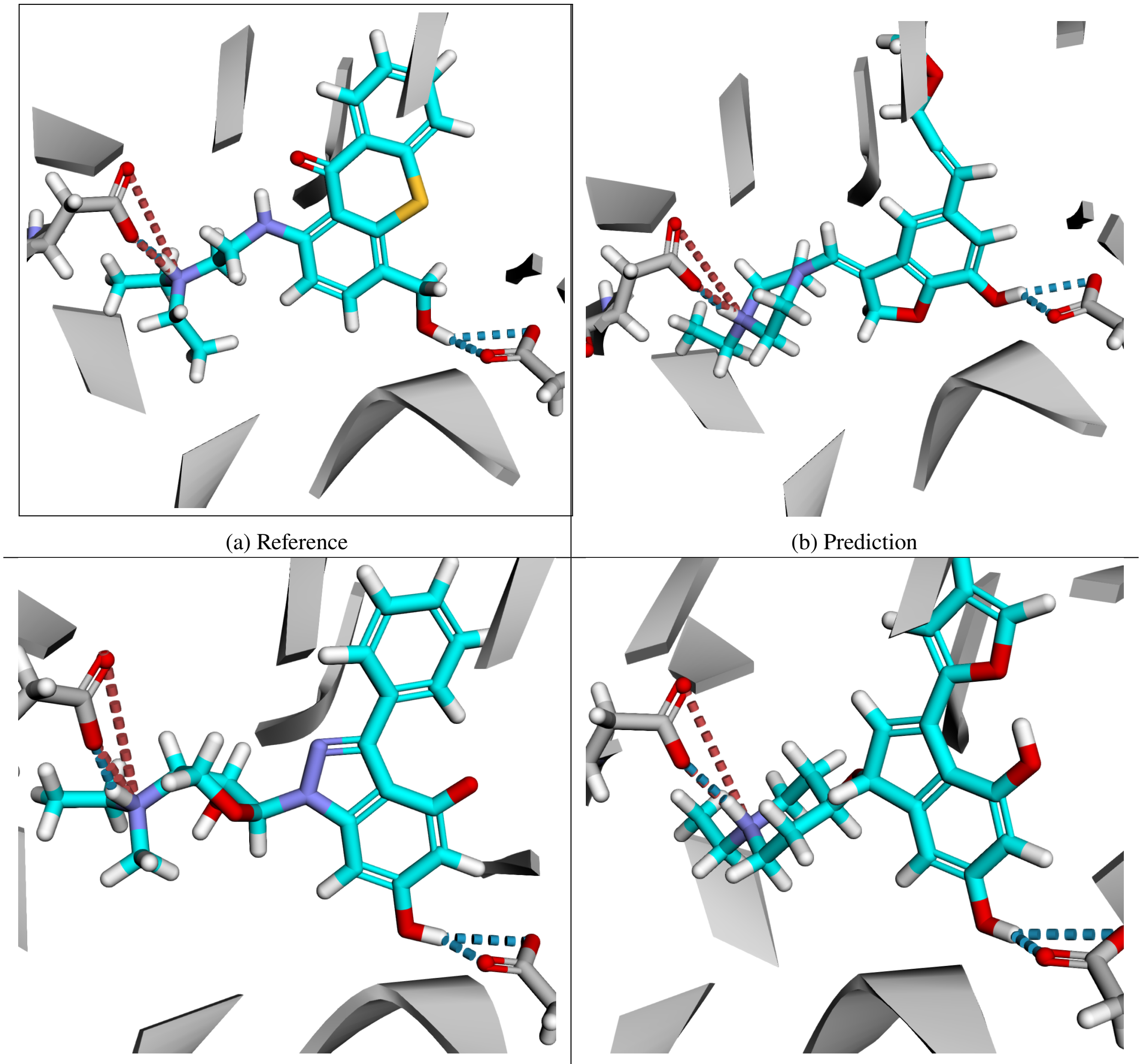

(a) Reference (b) Prediction (c) Prediction (d) Prediction

Supplementary Figure 6: Comparison of reference and predicted ligands on their interaction profiles for the pocket of the protein with PDB id 6UUX sampled with FLOWR.MULTI. Atom colors: C (cyan/gray), N (blue), O (red), S (yellow), F (ochre), Cl (green), H (white)

Supplementary Table 6: **Evaluation of FLOWR.MULTI on 4MPE.** Performance evaluation for interaction-, scaffold-, and functional group-conditional generation with FLOWR.MULTI on the test target with PDB-ID 4MPE. We report PoseBusters-validity (PB-validity) across 100 ligands per target, the mean Vina score (kcal/mol) as well as interaction recovery rate (PLIF recovery) and synthesizability score (SA score).

PROTEIN	METRIC	REFERENCE	FLOWR.MULTI$^{\text{interact.-cond.}}$	FLOWR.MULTI$^{\text{scaffold-cond.}}$	FLOWR.MULTI$^{\text{f. group– cond.}}$
4MPE	PB-VALIDITY ↑	1.0	0.95	1.0	0.92
	VINA SCORE ↓	-7.23	-6.80	-7.27	-6.41
	VINA SCORE (TOP-10) ↓	-	-7.54	-7.83	-7.15
	PLIF RECOVERY RATE ↑	-	0.79	0.53	0.89
	SA SCORE ↑	0.84	0.81	0.82	0.82

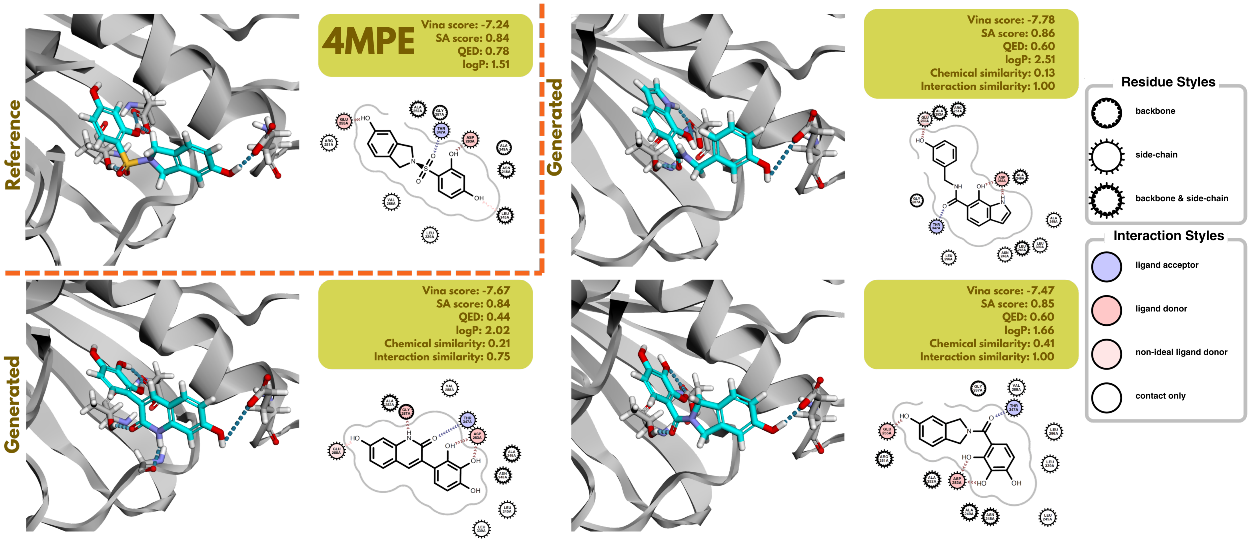

Supplementary Figure 7: **Evaluation of interaction-conditional generation on 4MPE with FLOWR.MULTI** Using the interaction-conditional generation mode of FLOWR.MULTI, we sample 100 ligands for a randomly selected target from the SPINDR test set, here 4MPE. We then select three ligands at random and compare them to the reference compound based on Vina score, SA score, QED, logP, chemical similarity, and interaction similarity. Atom colors: C (cyan/gray), N (blue), O (red), S (yellow), F (ochre), Cl (green), H (white)

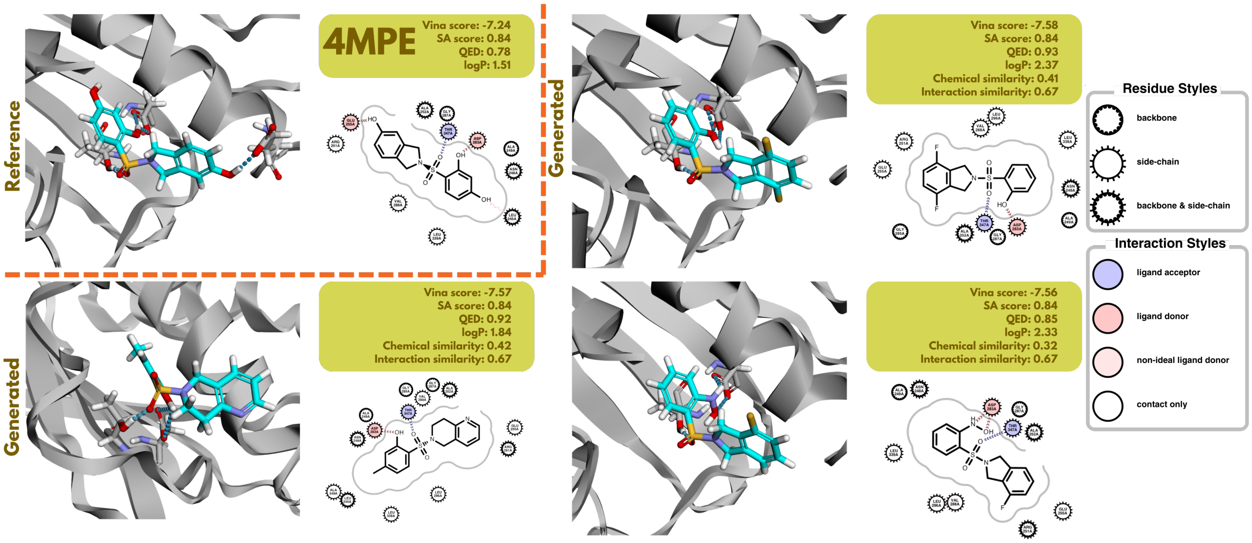

Supplementary Figure 8: **Evaluation of scaffold-conditional generation on 4MPE with FLOWR.MULTI** Using the scaffold-conditional generation mode of FLOWR.MULTI, we sample 100 ligands for a randomly selected target from the SPINDR test set, here 4MPE. We then select three ligands at random and compare them to the reference compound based on Vina score, SA score, QED, logP, chemical similarity, and interaction similarity. Atom colors: C (cyan/gray), N (blue), O (red), S (yellow), F (ochre), Cl (green), H (white)

2.6 4MPE: Visualizations

Here we show additional results on the protein target with PDB ID 4MPE for different conditional modes using the FLOWR.MULTI model.

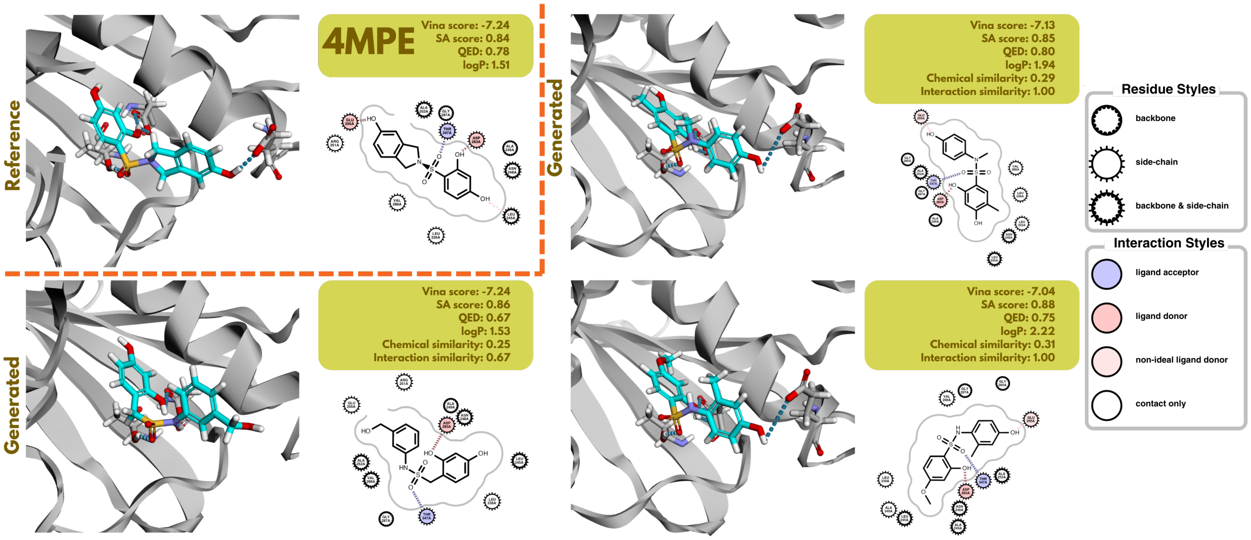

Supplementary Figure 9: **Evaluation of functional-group-conditional generation on 4MPE with FLOWR.MULTI** Using the functional-group-conditional generation mode of FLOWR.MULTI, we sample 100 ligands for a randomly selected target from the SPINDR test set, here 4MPE. We then select three ligands at random and compare them to the reference compound based on Vina score, SA score, QED, logP, chemical similarity, and interaction similarity. Atom colors: C (cyan/gray), N (blue), O (red), S (yellow), F (ochre), Cl (green), H (white)

Supplementary Table 7: **Evaluation of FLOWR.MULTI on 5YEA.** Performance evaluation for interaction-, scaffold-, and functional group-conditional generation with FLOWR.MULTI on a randomly selected test target with PDB-ID 5YEA. We report PoseBusters-validity (PB-validity) across 100 ligands per target, the mean Vina score (kcal/mol) as well as interaction recovery rate (PLIF recovery) and synthesizability score (SA score).

PROTEIN	METRIC	REFERENCE	FLOWR.MULTI$^{\text{interact.-cond.}}$	FLOWR.MULTI$^{\text{scaffold-cond.}}$	FLOWR.MULTI$^{\text{f. group-cond.}}$
5YEA	PB-VALIDITY ↑	1.0	0.90	0.98	0.89
	VINA SCORE ↓	-9.57	-8.96	-8.71	-8.99
	VINA SCORE (TOP-10) ↓	-	-10.08	-10.16	-8.99
	PLIF RECOVERY RATE ↑	-	0.87	0.75	0.77
	SA SCORE ↑	0.82	0.77	0.82	0.76

2.7 5YEA: Visualizations

Here we show additional results on the protein target with PDB ID 5YEA for different conditional modes using FLOWR.MULTI.

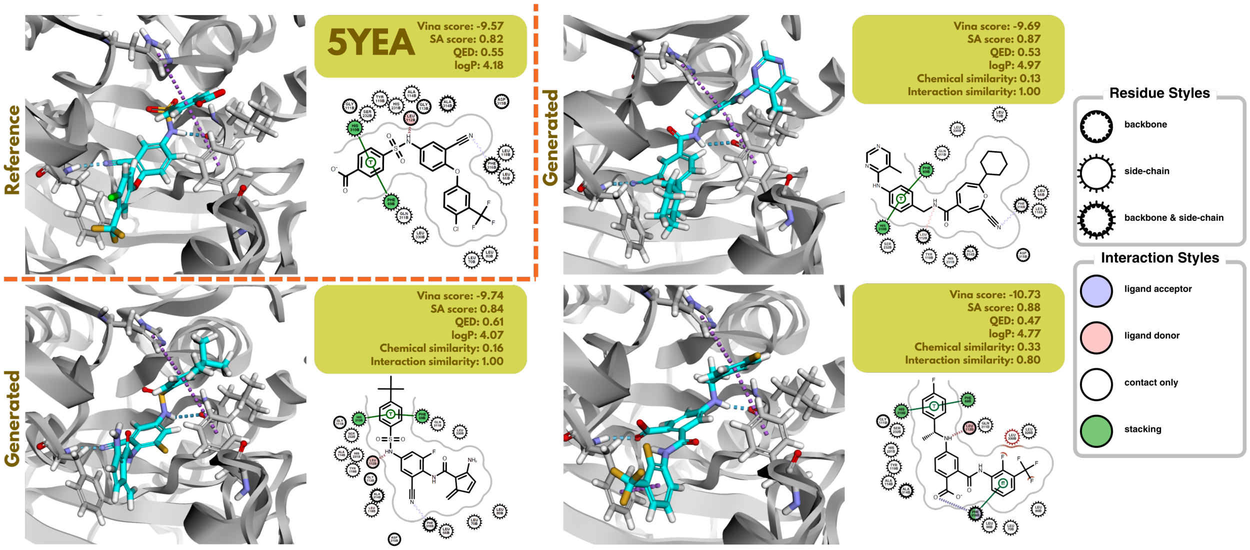

Supplementary Figure 10: **Evaluation of interaction-conditional generation on 5YEA with FLOWR.MULTI** Using the interaction-conditional generation mode of FLOWR.MULTI, we sample 100 ligands for a randomly selected target from the SPINDR test set, here 5YEA. We then select three ligands at random and compare them to the reference compound based on Vina score, SA score, QED, logP, chemical similarity, and interaction similarity.

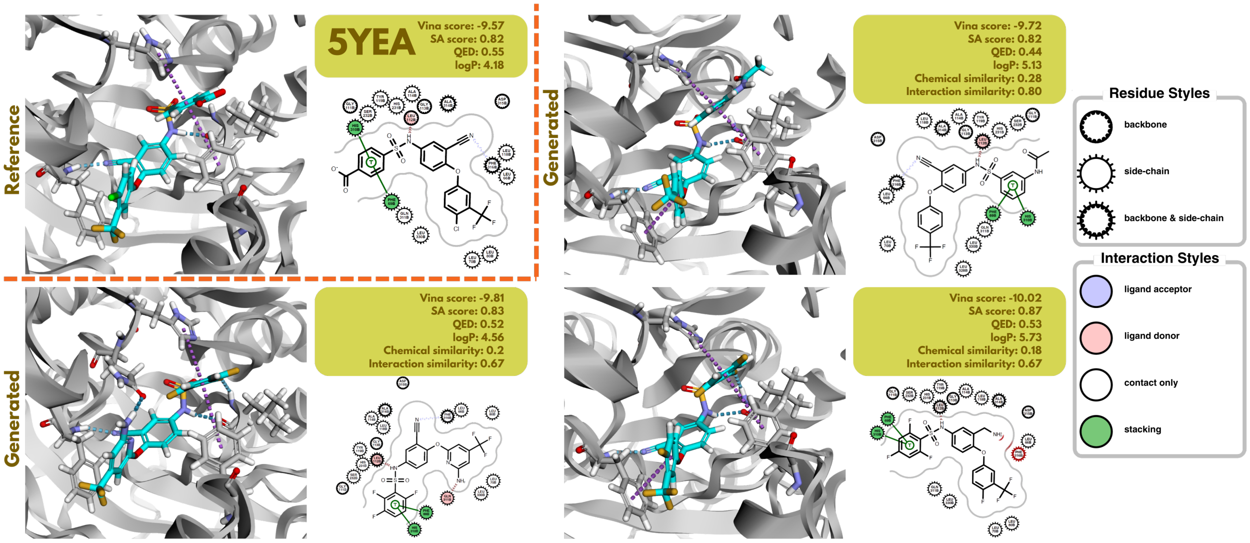

Supplementary Figure 11: **Evaluation of scaffold-conditional generation on 5YEA with FLOWR.MULTI** Using the scaffold-conditional generation mode of FLOWR.MULTI, we sample 100 ligands for a randomly selected target from the SPINDR test set, here 5YEA. We then select three ligands at random and compare them to the reference compound based on Vina score, SA score, QED, logP, chemical similarity, and interaction similarity. Atom colors: C (cyan/gray), N (blue), O (red), S (yellow), F (ochre), Cl (green), H (white)

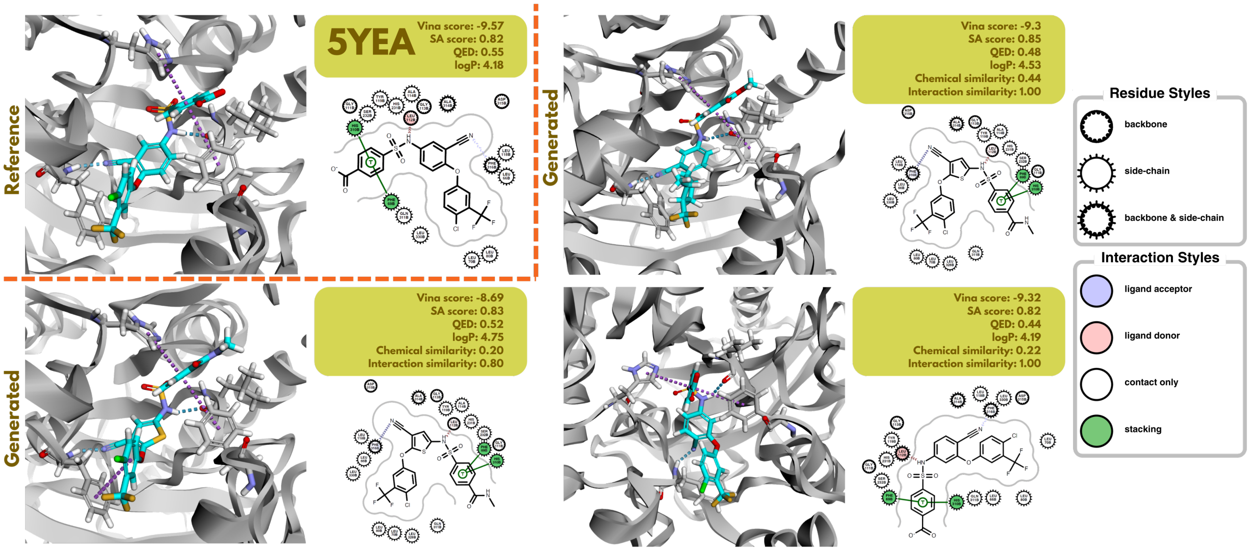

Supplementary Figure 12: **Evaluation of functional-group-conditional generation on 5YEA with FLOWR.MULTI** Using the functional-group-conditional generation mode of FLOWR.MULTI, we sample 100 ligands for a randomly selected target from the SPINDR test set, here 5YEA. We then select three ligands at random and compare them to the reference compound based on Vina score, SA score, QED, logP, chemical similarity, and interaction similarity. Atom colors: C (cyan/gray), N (blue), O (red), S (yellow), F (ochre), Cl (green), H (white)

References

[1] Benoit Baillif, Jason Cole, Patrick McCabe, and Andreas Bender. Benchmarking structure-based three-dimensional molecular generative models using genbench3d: ligand conformation quality matters, 2024. URL `https://arxiv.org/abs/2407.04424`.

[2] Christoph Bannwarth, Sebastian Ehlert, and Stefan Grimme. Gfn2-xtb—an accurate and broadly parametrized self-consistent tight-binding quantum chemical method with multipole electrostatics and density-dependent dispersion contributions. *Journal of Chemical Theory and Computation*, 15(3):1652–1671, Mar 2019. ISSN 1549-9618. doi: 10.1021/acs.jctc.8b01176. URL `https://doi.org/10.1021/acs.jctc.8b01176`.

[3] Cédric Bouysset and Sébastien Fiorucci. Prolif: a library to encode molecular interactions as fingerprints. *Journal of Cheminformatics*, 13(1):72, Sep 2021. ISSN 1758-2946. doi: 10.1186/s13321-021-00548-6. URL `https://doi.org/10.1186/s13321-021-00548-6`.

[4] Sebastian Ehlert, Marcel Stahn, Sebastian Spicher, and Stefan Grimme. Robust and efficient implicit solvation model for fast semiempirical methods. *Journal of Chemical Theory and Computation*, 17(7):4250–4261, Jul 2021. ISSN 1549-9618. doi: 10.1021/acs.jctc.1c00471. URL `https://doi.org/10.1021/acs.jctc.1c00471`.

[5] David Errington, Constantin Schneider, Cédric Bouysset, and Frédéric A. Dreyer. Assessing interaction recovery of predicted protein-ligand poses, 2024. URL `https://arxiv.org/abs/2409.20227`.

[6] Peter Ertl and Ansgar Schuffenhauer. Estimation of synthetic accessibility score of drug-like molecules based on molecular complexity and fragment contributions. *Journal of cheminformatics*, 1(1):1–11, 2009.

[7] W Patrick Walters, Ajay A Murcko, and Mark A Murcko. Recognizing molecules with drug-like properties. *Current Opinion in Chemical Biology*, 3(4):384–387, 1999. ISSN 1367-5931. doi: https://doi.org/10.1016/S1367-5931(99)80058-1. URL `https://www.sciencedirect.com/science/article/pii/S1367593199800581`.
