# Supplementary material for: FLOWR: flow matching for structure-aware de novo, interaction- and fragment-based ligand generation
Source: Nat Comput Sci. 2026 May 28;6(6):565–74. doi: 10.1038/s43588-026-00998-8 (PMC13293864; doi:10.1038/s43588-026-00998-8)
Supplement: Supplementary file 3 — Peer Review File [file 43588_2026_998_MOESM3_ESM.pdf]

Peer Review File

FLOWR: Flow Matching for Structure-Aware De Novo, Interaction- and Fragment-Based Ligand Generation

Corresponding Author: Dr Julian Cremer

This file contains all editorial decision letters in order by version, followed by all author rebuttals in order by version.

Version 0:

Decision Letter:

** Please ensure you delete the link to your author homepage in this e-mail if you wish to forward it to your co-authors. **

Dear Dr Cremer,

Thank you again for submitting your manuscript "FLOWR: Flow Matching for Structure-Aware \textit{De Novo}, Interaction- and Fragment-Based Ligand Generation”. I am pleased to tell you that we are sending your paper out for formal peer review. Before we can do so, please read the below carefully as we require a few documents.

If you have not done so already, please alert us to any related manuscripts from your group that are under consideration or in press at other journals, or are being written up for submission to other journals (see https://www.nature.com/authors/policies/duplicate.html for details).

We are asking all corresponding authors of primary research articles to complete an Editorial Policy Checklist that verifies compliance with all required editorial policies. The form should be completed and returned within 48 hours, if you have not already done so. Please note that the form is a dynamic 'smart pdf' and must therefore be downloaded and completed in Adobe Reader. We will then flatten them for ease of use by the reviewers. If you would like to reference the guidance text as you complete the template, please access these flattened versions at https://www.nature.com/authors/policies/availability.html

Editorial Policy Checklist: https://www.nature.com/documents/nr-editorial-policy-checklist.pdf

We also ask that you complete a reporting summary that collects information on experimental design:
Reporting summary: https://www.nature.com/documents/nr-reporting-summary.pdf

In addition, as your paper relies on code that is central to the main claims, it should be shared with the referees during the peer review process and publicly at least on publication. If there are restrictions to public sharing on publication, please discuss them with us.

We would like to ask you that you please provide a link to a code repository and complete the following software and custom code submission checklist:

Software supplement: https://www.nature.com/documents/nr-software-policy.pdf
(Please note that the form is a dynamic 'smart pdf' and must therefore be downloaded and completed in Adobe Reader.)

Alternatively, or as an additional resource, please consider setting up an executable compute capsule for your code on the cloud-based platform Code Ocean, to facilitate the review process as well as the reproducibility and re-usability of your code. Code Ocean staff will assist you in generating a compute capsule for your code and provide a working copy of this compute capsule for use in the peer review process (after a brief review by Code Ocean staff, to ensure that everything runs). If you have selected Double Blind Peer Review, we will make sure the capsule contains no information about your identity.

Please let us know as soon as possible if you wish to participate and we will provide you with further information about setting up a compute capsule. An overview of the process can be found here: <a href="https://help.codeocean.com/publishing-on-code-ocean/peer-review-on-code-

ocean">https://help.codeocean.com/publishing-on-code-ocean/peer-review-on-code-ocean.

If you do not opt for setting a Code Ocean capsule, please fill in the software and custom code submission checklist.

Some further information about using Code Ocean for peer review and publication of code can be found in this blog post https://help.codeocean.com/en/articles/2644233-peer-review-on-code-ocean and in Code Ocean's user guide https://docs.codeocean.com/user-guide/.

To improve transparency in authorship we are requesting that all authors identified as ‘corresponding author’ on published papers create and link their Open Researcher and Contributor Identifier (ORCID) with their account on the Manuscript Tracking System (MTS), prior to acceptance. ORCID helps the scientific community achieve unambiguous attribution of all scholarly contributions. You can create and link your ORCID from the home page of the MTS by clicking on ‘Modify my Springer Nature account’. For more information please visit please visit www.springernature.com/orcid.

Finally, we encourage you to share a preprint of the original submitted version of your paper so as to minimize delays in communicating your research findings; benefits of preprints include early visibility, and citations (https://www.natureindex.com/news-blog/preprints-boost-article-citations-and-mentions) and demonstration of research progress. You may want to consider the multidisciplinary Research Square preprint platform (https://www.researchsquare.com/browse), provided by our partner Research Square, where your preprint will be publicly available with a citable DOI under a CC-BY license. You are of course free to use a discipline-specific preprint platform of your choice. More information about our preprint policy can be found in the following link: https://www.nature.com/nature-research/editorial-policies/preprints-and-conference-proceedings#preprints

Please use the following link to submit the required checklists; please also resubmit your original manuscript files, or revised versions of them as a result of filling out the checklists:

Link Redacted

** This url links to your confidential homepage and associated information about manuscripts you may have submitted or be reviewing for us. If you wish to forward this e-mail to co-authors, please delete this link to your homepage first. **

Please note that Nature Computational Science implements transparent peer review of original research manuscripts, in which we publish the reviewer comments to the authors, author rebuttal letters and editorial decision letters as a supplementary peer review file, if the author agrees at the point of acceptance. This will apply to new manuscripts submitted on or after 17th Feb 2021. Upon author request, confidential information and data can be removed from the reviewer reports and rebuttal letters prior to publication. For more information, please refer to our FAQ page.

Best regards,

Kaitlin McCardle, PhD
Senior Editor
Nature Computational Science

Version 1:

Decision Letter:

** Please ensure you delete the link to your author homepage in this e-mail if you wish to forward it to your co-authors. **

Dear Dr Cremer,

Your manuscript "FLOWR: Flow Matching for Structure-Aware De Novo, Interaction- and Fragment-Based Ligand Generation" has now been seen by 2 referees, whose comments are appended below. You will see that while they find your work of interest, they have raised points that need to be addressed before we can make a decision on publication.

The referees’ reports seem to be quite clear. Naturally, we will need you to address *all* of the points raised.

While we ask you to address all of the points raised, the following points need to be substantially worked on:

1) Please provide additional comparisons and methodological justifications
2) Please be sure to demonstrate that the generated molecules are practically useful

Please use the following link to submit your revised manuscript and a point-by-point response to the referees’ comments (which should be in a separate document to any cover letter):

Link Redacted

** This url links to your confidential homepage and associated information about manuscripts you may have submitted or be reviewing for us. If you wish to forward this e-mail to co-authors, please delete this link to your homepage first. **

To aid in the review process, we would appreciate it if you could also provide a copy of your manuscript files that indicates your revisions by making use of Track Changes or similar mark-up tools. Please also ensure that all correspondence is marked with your Nature Computational Science reference number in the subject line.

In addition, please make sure to upload a Word Document or LaTeX version of your text, to assist us in the editorial stage.

To improve transparency in authorship, we request that all authors identified as 'corresponding author' on published papers create and link their Open Researcher and Contributor Identifier (ORCID) with their account on the Manuscript Tracking System (MTS), prior to acceptance. ORCID helps the scientific community achieve unambiguous attribution of all scholarly contributions. You can create and link your ORCID from the home page of the MTS by clicking on 'Modify my Springer Nature account'. For more information please visit please visit www.springernature.com/orcid.

We hope to receive your revised paper within three weeks. If you cannot send it within this time, please let us know.

We look forward to hearing from you soon.

Best regards,

Kaitlin McCardle, PhD
Senior Editor
Nature Computational Science

Reviewers comments:

Reviewer #1 (Remarks to the Author):

This manuscript introduces FLOWR, a novel flow matching-based generative model for structure-based drug discovery (SBDD), integrating equivariant optimal transport and efficient pocket conditioning to generate 3D ligands. It also presents SPINDR, a curated dataset from PLINDER that addresses structural defects and data leakage. FLOWR.MULTI extends the model for versatile conditional generation, supporting interaction- and fragment-based design without retraining. Evaluations on CROSSDOCKED2020 and SPINDR demonstrate superior performance over state-of-the-art methods in validity, pose accuracy, interaction recovery, and inference speed, marking a significant advance in AI-driven SBDD for de novo design, hit expansion, and fragment-based optimization. Here are some questions and suggestion:
1. There is a significant decrease in validity metrics (e.g., PoseBusters-validity from 0.88 to 0.60) when explicit hydrogens are included in training and inference, what is the reason? Furthermore, it does not explain the reversal in strain energy performance compared to PILOT (better without hydrogens at 90.05 vs. 120.10 kcal/mol, but worse with hydrogens at 54.11 vs. 53.07 kcal/mol).
2. For SPINDR, quantitative comparisons are limited to relaxation energies and substructures, omitting metrics like interaction diversity and pocket similarity, and external validation is absent, undermining claims of its superiority over CROSSDOCKED2020. I suggest expanding evaluations with broader metrics and retraining additional models (e.g., retraining models like TARGETDIFF or DIFFSBDD on SPINDR) to demonstrate reduced biases and improved generalization.
3. SPINDR's smaller size (35k vs. CROSSDOCKED's ~100k) may lead to coverage gaps in protein families, pocket types, or ligand spaces, yet comparative analyses of these distributions (e.g., protein classes, pocket volumes, ligand scaffolds) are missing, potentially revealing biases affecting generalization. I suggest expanding comparative evaluations by conducting detailed analyses of dataset distributions (e.g., protein classes and ligand scaffolds) in comparison to CROSSDOCKED2020.
4. The E(3)-equivariant architecture preserves rotational and translational invariance but does not explicitly handle chirality or higher-order symmetries, which could cause inaccuracies in generating stereochemically complex ligands, especially in SBDD where enantiomers differ in binding affinity. I suggest clarifying this limitation and exploring extensions for chirality handling.
5. Lacks discussions on broader impacts, such as limitations and future work.
6. Lacks motivations for equivariant optimal transport and details of fragmentation strategies.
7. Lacks ablation studies to evaluate the impact of latent layer in the cross-attention module on model accuracy and efficiency.

Reviewer #2 (Remarks to the Author):

The authors here present FLOWR, a novel generative framework for structure-based ligand design, which the authors claim provides significant advantages in both sample quality and computational efficiency over existing diffusion-based models. A

key contribution of this work is the introduction of a new curated dataset named SPINDR, designed to address the structure quality issues prevalent in the earlier reported PLINDER dataset. The paper evaluates FLOWR against state-of-the-art models, including a recent diffusion model, PILOT, and demonstrates superior performance across a range of metrics including PoseBusters-validity, strain energy, and interaction recovery, while being up to 70-fold faster. Furthermore, the authors introduce FLOWR.MULTI, an extension capable of multi-purpose conditional generation (e.g., fragment-based, interaction-conditional) without requiring model retraining or expensive resampling strategies. The work appears a worthy contribution to this fast moving research area, and this reviewer would recommend publication with minor revisions.

The two areas where this reviewer believe the manuscript might be further improved are the following:

(1) I was surprised by the following line of the paper: "Data deduplication. Since existing datasets often contain significant structural redundancy, we also experiment with two data deduplication strategies – we present these in more detail in Appendix A. For the remainder of this paper, however, we make use of the non-deduplicated version of SPINDR." This choice seems surprising, and more discussion in the main text of the paper is warranted. A clear justification for the choice was also not apparent to this reader after reviewing the material in the Appendix. Some additional clarity around this methodological choice in the main text of the manuscript would benefit the reader.

(2) The authors rightly note that the strain energies of generated ligands from both FLOWR (90.05 kcal/mol) and PILOT (120.10 kcal/mol) are substantially higher than that of the test set (43.27 kcal/mol). While they hypothesize this is due to limited training data coverage, this is a significant limitation regarding the physical realism of the generated molecules. This point warrants more discussion. This reviewer could not get a clear sense from the text regarding whether or not the molecules looked 'mangled' versus what one would expect to see from an experimental crystal structure, or even a high-quality docked pose using traditional physics-based docking methods. Would these strained molecules be easily relaxed using local minimization with a classical force field? Exploring these topics in greater detail would be beneficial to the work.

Version 2:

Decision Letter:

Our ref: NATCOMPUTSCI-25-1299B

4th December 2025

Dear Dr. Cremer,

Thank you for submitting your revised manuscript "FLOWR: Flow Matching for Structure-Aware De Novo, Interaction- and Fragment-Based Ligand Generation" (NATCOMPUTSCI-25-1299B). It has now been seen by the original referees and their comments are below. The reviewers find that the paper has improved in revision, and therefore we'll be happy in principle to publish it in Nature Computational Science, pending minor revisions to satisfy the referees' final requests and to comply with our editorial and formatting guidelines.

We are now performing detailed checks on your paper and will send you a checklist detailing our editorial and formatting requirements in the next few weeks. Please do not upload the final materials and make any revisions until you receive this additional information from us.

TRANSPARENT PEER REVIEW
Nature Computational Science offers a transparent peer review option for original research manuscripts. We encourage increased transparency in peer review by publishing the reviewer comments, author rebuttal letters and editorial decision letters if the authors agree. Such peer review material is made available as a supplementary peer review file. **Please remember to choose, using the manuscript system, whether or not you want to participate in transparent peer review.**
Please note: we allow redactions to authors' rebuttal and reviewer comments in the interest of confidentiality. If you are concerned about the release of confidential data, please let us know specifically what information you would like to have removed. Please note that we cannot incorporate redactions for any other reasons. Reviewer names will be published in the peer review files if the reviewer signed the comments to authors, or if reviewers explicitly agree to release their name. For more information, please refer to our FAQ page.

Thank you again for your interest in Nature Computational Science. Please do not hesitate to contact me if you have any questions.

Sincerely,

Kaitlin McCardle, PhD
Senior Editor
Nature Computational Science

ORCID

IMPORTANT: Non-corresponding authors do not have to link their ORCIDs but are encouraged to do so. Please note that it will not be possible to add/modify ORCIDs at proof. Thus, please let your co-authors know that if they wish to have their ORCID added to the paper they must follow the procedure described in the following link prior to acceptance: https://www.springernature.com/gp/researchers/orcid/orcid-for-nature-research

Author names using non-Roman characters
Nature Portfolio journals can support presentation of author names using non-Roman characters in the HTML version of the article. If you wish to, please include author names in parentheses after the Roman-character spelling; see example online here. Currently supported scripts are: Arabic, Chinese, Cyrillic, Devanagari, Greek, Hebrew, Hangul, Japanese and Persian. You will be asked to verify the rendering is correct at proof stage.

Reviewer #1 (Remarks to the Author):

I have no further comments.

Reviewer #2 (Remarks to the Author):

This reviewer thanks the authors for their efforts and believes the revised manuscript is now suitable for publication.

Version 3:

Decision Letter:

Dear Dr Cremer,

We are pleased to inform you that your Brief Communication "FLOWR: Flow Matching for Structure-Aware De Novo, Interaction- and Fragment-Based Ligand Generation" has now been accepted for publication in Nature Computational Science.

Once your manuscript is typeset, you will receive an email with a link to choose the appropriate publishing options for your paper and our Author Services team will be in touch regarding any additional information that may be required.

Authors may need to take specific actions to achieve compliance with funder and institutional open access mandates. If your research is supported by a funder that requires immediate open access (e.g. according to Plan S principles or the NIH public access policy) then you should select the gold OA route, and we will direct you to the compliant route where possible. Because authors warrant under our subscription licensing terms that they haven't committed to licensing any version of their article under a licence inconsistent with the terms of our agreement – including the applicable embargo period – publication under the subscription model isn't suitable for authors whose funders require no embargo.

If you have any questions about our publishing options, costs, Open Access requirements, or our legal forms, please contact ASJournals@springernature.com

Acceptance of your manuscript is conditional on all authors' agreement with our publication policies (see https://www.nature.com/natcomputsci/for-authors). In particular your manuscript must not be published elsewhere and there must be no announcement of the work to any media outlet until the publication date (the day on which it is uploaded onto our web site).

Before your manuscript is typeset, we will edit the text to ensure it is intelligible to our wide readership and conforms to house style. We look particularly carefully at the titles of all papers to ensure that they are relatively brief and understandable.

Once your manuscript is typeset, you will receive a link to your electronic proof via email with a request to make any corrections within 48 hours. If, when you receive your proof, you cannot meet this deadline, please inform us at rjsproduction@springernature.com immediately.

If you have queries at any point during the production process then please contact the production team at rjsproduction@springernature.com.

You may wish to make your media relations office aware of your accepted publication, in case they consider it appropriate to organize some internal or external publicity. Once your paper has been scheduled you will receive an email confirming the publication details. This is normally 3-4 working days in advance of publication. If you need additional notice of the date and time of publication, please let the production team know when you receive the proof of your article to ensure there is sufficient time to coordinate. Further information on our embargo policies can be found here: https://www.nature.com/authors/policies/embargo.html

An online order form for reprints of your paper is available at https://www.nature.com/reprints/author-reprints.html. All co-authors, authors' institutions and authors' funding agencies can order reprints using the form appropriate to their geographical region.

We welcome the submission of potential cover material (including a short caption of around 40 words) related to your manuscript; suggestions should be sent to Nature Computational Science as electronic files (the image should be 300 dpi at 210 x 297 mm in either TIFF or JPEG format). We also welcome suggestions for the Hero Image, which appears at the top of our home page; these should be 72 dpi at 1400 x 400 pixels in JPEG format. Please note that such pictures should be selected more for their aesthetic appeal than for their scientific content, and that colour images work better than black and white or grayscale images. Please do not try to design a cover with the Nature Computational Science logo etc., and please do not submit composites of images related to your work. I am sure you will understand that we cannot make any promise as to whether any of your suggestions might be selected for the cover of the journal.

You can now use a single sign-on for all your accounts, view the status of all your manuscript submissions and reviews, access usage statistics for your published articles and download a record of your refereeing activity for the Nature journals.

To assist our authors in disseminating their research to the broader community, our SharedIt initiative provides you with a unique shareable link that will allow anyone (with or without a subscription) to read the published article. Recipients of the link with a subscription will also be able to download and print the PDF.

As soon as your article is published, you will receive an automated email with your shareable link.

We look forward to publishing your paper.

Best regards,

Kaitlin McCardle, PhD
Senior Editor
Nature Computational Science

P.S. Click on the following link if you would like to recommend Nature Computational Science to your librarian: https://www.springernature.com/gp/librarians/recommend-to-your-library

** Visit the Springer Nature Editorial and Publishing website at www.springernature.com/editorial-and-publishing-jobs for more information about our career opportunities. If you have any questions please click here.**

Open Access This Peer Review File is licensed under a Creative Commons Attribution 4.0 International License, which permits use, sharing, adaptation, distribution and reproduction in any medium or format, as long as you give appropriate

credit to the original author(s) and the source, provide a link to the Creative Commons license, and indicate if changes were made.
In cases where reviewers are anonymous, credit should be given to 'Anonymous Referee' and the source.
The images or other third party material in this Peer Review File are included in the article's Creative Commons license, unless indicated otherwise in a credit line to the material. If material is not included in the article's Creative Commons license and your intended use is not permitted by statutory regulation or exceeds the permitted use, you will need to obtain permission directly from the copyright holder.
To view a copy of this license, visit https://creativecommons.org/licenses/by/4.0/

Response to Reviewers

Nature Computational Science
Manuscript NATCOMPUTSCI-25-1299A

FLOWR – Flow Matching for Structure-Aware *De Novo*, Interaction- and Fragment-Based Ligand Generation

---

Julian Cremer[1,*,†], **Ross Irwin**[2,3,*,†], **Alessandro Tibo**[2], **Jon Paul Janet**[2], **Simon Olsson**[3], **Djork-Arné Clevert**[1]

[1]Machine Learning & Computational Sciences, Pfizer Worldwide R&D, Berlin, Germany
[2]Molecular AI, Discovery Sciences, R&D, AstraZeneca, Gothenburg, Sweden
[3]Department of Computer Science and Engineering,
Chalmers University of Technology and University of Gothenburg, Gothenburg, Sweden

[*]Corresponding authors. [†]These authors contributed equally to this work.

Dear Editor and Reviewers,

We thank the reviewers for their thoughtful and constructive feedback on our manuscript. We appreciate their recognition of FLOWR's contributions to structure-based drug discovery and the value of the SPINDR dataset. Below, we provide detailed responses to each comment raised by the reviewers. All changes to the manuscript are highlighted in red.

Contents

1 Response to Reviewer #1 **2**
1.1 Comment 1.1: Validity metrics with explicit hydrogens 2
1.2 Comment 1.2: SPINDR quantitative comparisons and external validation 2
1.3 Comment 1.3: SPINDR dataset size and coverage 3
1.4 Comment 1.4: Chirality and stereochemistry handling 4
1.5 Comment 1.5: Broader impacts, limitations, and future work 4
1.6 Comment 1.6: Motivations for equivariant optimal transport and fragmentation strategies 5
1.7 Comment 1.7: Ablation studies on cross-attention module 6

2 Response to Reviewer #2 **7**
2.1 Comment 2.1: Data deduplication strategy justification 7
2.2 Comment 2.2: Strain energy analysis and physical realism 7

1 Response to Reviewer #1

We thank Reviewer #1 for their positive assessment of FLOWR and their valuable suggestions for improving the manuscript. We address each point below.

1.1 Comment 1.1: Validity metrics with explicit hydrogens

Reviewer Comment: There is a significant decrease in validity metrics (e.g., PoseBusters-validity from 0.88 to 0.60) when explicit hydrogens are included in training and inference, what is the reason? Furthermore, it does not explain the reversal in strain energy performance compared to PILOT (better without hydrogens at 90.05 vs. 120.10 kcal/mol, but worse with hydrogens at 54.11 vs. 53.07 kcal/mol).

Our Response: Modeling explicit hydrogens alongside heavy atoms substantially increases task and learning complexity. Although the SPINDR dataset offers notable improvements in data quality and dataset splitting over existing SBDD datasets, it still provides limited coverage of chemical and conformational space. Models trained on larger datasets, such as GEOM-Drugs (300,000 molecules, >5 million conformers), achieve near-perfect PoseBusters validity scores, suggesting that reduced performance on SPINDR primarily reflects data scarcity.

Despite lower overall validity scores, strain energy metrics show significant improvement when modeling explicit hydrogens. This improvement has two explanations: First, heavy-atom-only models require post-hoc hydrogen addition via RDKit with random coordinates, artificially inflating strain energies. Second and most importantly, although learning complexity increases substantially, explicit hydrogen modeling yields superior performance in bond length, bond angle, and dihedral angle distributions. Notably, the reduced PoseBusters validity stems predominantly from ligand-protein clashes (minimum distance to protein metric), while internal energy evaluations converge to values comparable with heavy-atom models.

We anticipate that ligand-protein clashes could be reduced substantially under two conditions: (1) increased training data with broader chemical and conformational coverage, and (2) explicit hydrogen modeling in protein pockets. While our model supports this in principle, computational requirements for this ($\sim$ 2000 GPU-hours on 80GB GPUs) render it infeasible for small- to medium-sized research laboratories.

However, we want to emphasize that future models should be evaluated on their accuracy in explicit hydrogen modeling and their ability to scale efficiently beyond merely increasing computational resources - and to this end, the SPINDR dataset provides the requisite high-quality benchmark and our model establishes an initial baseline for such evaluations.

Changes to manuscript: We added a concise version of this explanation to the main text.

1.2 Comment 1.2: SPINDR quantitative comparisons and external validation

Reviewer Comment: For SPINDR, quantitative comparisons are limited to relaxation energies and substructures, omitting metrics like interaction diversity and pocket similarity, and external validation is absent, undermining claims of its superiority over CROSSDOCKED2020. I suggest expanding evaluations with broader metrics and retraining additional models (e.g., retraining models like TARGETDIFF or DIFFSBDD on SPINDR) to demonstrate reduced biases and improved generalization.

Our Response:
While both datasets derive from the PDB and therefore exhibit comparable target family coverage, pocket similarity, and chemical space coverage, SPINDR offers critical advantages in data quality and experimental design. First, SPINDR complexes undergo rigorous preprocessing using Schrödinger's PrepWizard, including proper protonation state assignment and structural relaxation, followed by filtering for drug-likeness and redundancy. Second, SPINDR strictly adheres to the Plinder dataset split methodology, which represents the most rigorous train/validation/test splitting approach to date, ensuring proper evaluation of model generalization. Third, we provide ProLIF-derived interaction fingerprint annotations for all complexes, enabling systematic interaction recovery evaluation.
In contrast, CrossDocked2020 relies on low-level-of-theory rigid pocket cross-docking using PDBBind-derived structures, yielding computationally generated poses that often exhibit unrealistic geometries and strain. Critically, SPINDR contains exclusively experimental co-crystal structures rather than docked poses, providing ground-truth conformations with direct relevance to drug discovery applications. These fundamental differences in data provenance and quality constitute clear superiority.
Regarding retraining existing models on SPINDR: We evaluated TargetDiff and DiffSBDD on CrossDocked2020, where both models perform substantially worse than subsequent methods, with PILOT and particularly FLOWR demonstrating clear superiority. Notably, data augmentation via cross-docking provides a larger dataset with a more easily learned distribution, albeit at the cost of chemical realism. Given that earlier methods already underperform on this less challenging benchmark, we anticipate limited insights from retraining them on the more rigorous SPINDR dataset. Therefore, to minimize computational expense while maximizing scientific value, we selected PILOT – the second-best performing model—for comparative evaluation against FLOWR on SPINDR.

1.3 Comment 1.3: SPINDR dataset size and coverage

Reviewer Comment: SPINDR's smaller size (35k vs. CROSSDOCKED's ∼100k) may lead to coverage gaps in protein families, pocket types, or ligand spaces, yet comparative analyses of these distributions (e.g., protein classes, pocket volumes, ligand scaffolds) are missing, potentially revealing biases affecting generalization. I suggest expanding comparative evaluations by conducting detailed analyses of dataset distributions (e.g., protein classes and ligand scaffolds) in comparison to CROSSDOCKED2020.

Our Response:
Firstly, we stress that CrossDocked2020 is a synthetically-generated dataset created by cross-docking protein-ligand complexes in PDBBind. Both PDBBind and Spindr are derived from the PDB and so contain similar protein families and pocket layouts. In fact, we first extracted all reasonable systems from the PDB using PLINDER, and then filtered out systems with uninteresting ligands such as cofactors and ions. Secondly, we have now added a comparison of the uniqueness of ligands and ligand scaffolds between Spindr and CrossDocked2020 in Appendix A. Here we can see that, while CrossDocked2020 contains more ligands, it actually contains fewer unique ligands and significantly fewer unique scaffolds than Spindr. Ultimately, we see this as the advantage of taking data directly from the PDB – with our dataset as being more information rich than synthetically-generated datasets such as CrossDocked2020. Finally, beyond data diversity, we stress that one the key advantages of Spindr is the quality of the data. Generating data via rigid-pocket cross docking can create unrealistic ligand poses and strained conformations, as is shown in Appendix A.
Changes to manuscript: Added Table 7 in the Appendix to compare ligand diversity between Spindr and CrossDocked2020 and a discussion on the lower ligand diversity of CrossDocked2020.

1.4 Comment 1.4: Chirality and stereochemistry handling

Reviewer Comment: The E(3)-equivariant architecture preserves rotational and translational invariance but does not explicitly handle chirality or higher-order symmetries, which could cause inaccuracies in generating stereochemically complex ligands, especially in SBDD where enantiomers differ in binding affinity. I suggest clarifying this limitation and exploring extensions for chirality handling.

Our Response:
Our model explicitly incorporates protein-ligand interactions throughout the generative process via equivariant cross-attention between ligand and pocket encodings. Through this mechanism, the ligand decoder directly conditions on both invariant and equivariant pocket features, enabling stereochemical preferences to emerge naturally from learned geometric constraints. While the individual pocket encoder and ligand decoder maintain E(3)-equivariance, their cross-attention interaction produces chirality awareness by coupling ligand generation with pocket geometry, rendering separate chirality handling mechanisms unnecessary.

1.5 Comment 1.5: Broader impacts, limitations, and future work

Reviewer Comment: Lacks discussions on broader impacts, such as limitations and future work.

Our Response:
FLOWR addresses a critical challenge in early drug discovery by enabling structure-aware ligand generation for hit-to-lead and lead optimization campaigns. As an ideation tool, it can accelerate the exploration of chemically diverse binding modes and allows scaffold hopping as well as fragment growing and replacement strategies while maintaining geometric compatibility with target binding sites. By reducing reliance on extensive virtual screening or de novo design cycles, this approach has the potential to streamline drug development timelines and reduce associated costs. However, generated molecules require rigorous experimental validation, and the model should serve as a hypothesis generation tool rather than a replacement for medicinal chemistry expertise.
Current limitations include: (1) reduced validity when modeling explicit hydrogens due to limited training data and absence of protein-side hydrogen modeling; (2) computational requirements that may limit accessibility for smaller research groups; (3) evaluation primarily on the SPINDR benchmark, which, while high-quality, provides limited chemical and conformational space coverage compared to the full drug-like molecule landscape; and (4) reliance on static protein structures without accounting for conformational flexibility or induced fit effects.
Future directions include: (1) scaling to larger, more diverse datasets to improve explicit hydrogen modeling and with that reduce ligand-protein clashes; (2) incorporating protein pocket flexibility and explicit hydrogen modeling on the protein side; (3) integrating pharmacokinetic and synthetic accessibility constraints directly into the generative process; and (4) validating generated molecules through prospective experimental campaigns to assess real-world utility in drug discovery workflows.
Changes to manuscript: We added respective statements to the main text.

1.6 Comment 1.6: Motivations for equivariant optimal transport and fragmentation strategies

Reviewer Comment: Lacks motivations for equivariant optimal transport and details of fragmentation strategies.

Our Response:
Equivariant Optimal Transport: Our approach builds on continuous normalizing flows (CNFs) trained via conditional flow matching (CFM), which provides simulation-free training objectives combining the stable regression of diffusion models with efficient deterministic inference. Specifically, we employ optimal transport CFM (OT-CFM), which constructs simpler, more stable flows by minimizing transport costs between source and target distributions. We extend this framework with equivariant flow matching, exploiting the rotational and translational symmetries inherent to molecular systems for efficient training of equivariant CNFs. This combination yields flows with shorter integration paths, improved sampling efficiency, and incorporation of physical symmetries.
Fragmentation Strategies: We extract scaffolds using RDKit's `GetScaffoldForMol` from the MurckoScaffold implementation, with functional groups defined as all atoms not part of the scaffold and linkers as non-ring scaffold atoms. Additionally, we employ RDKit's matched molecular pairs analysis (rdMMPA) and its `FragmentMol` implementation to decompose molecules into chemically meaningful fragments, from which we randomly sample at each training batch. This enables the model to learn fragment-based generation strategies such as scaffold hopping and fragment growing. For interaction-conditional training, we utilize ProLIF-derived interaction fingerprints pre-calculated in the SPINDR dataset.
Changes to manuscript: We added an explanation regarding Equivariant Optimal Transport to the Appendix B (Additional Model Details), and added a section in the Appendix B about the fragmentation strategies (B.5).

1.7 Comment 1.7: Ablation studies on cross-attention module

Reviewer Comment: Lacks ablation studies to evaluate the impact of latent layer in the cross-attention module on model accuracy and efficiency.

Our Response:
There are no separate "latent layers" specific to the cross-attention module. Rather, FLOWR is fundamentally built on equivariant and invariant latent embeddings that serve as the foundation for both self-attention and cross-attention operations uniformly throughout the network. This unified representation strategy maintains architectural simplicity and computational efficiency while ensuring consistent geometric processing across all attention mechanisms. The cross-attention module operates on the same latent space as self-attention, enabling seamless protein-ligand interaction modeling without requiring additional architectural components or ablation analysis of module-specific layers.

2 Response to Reviewer #2

We thank Reviewer #2 for their positive evaluation and recommendation for publication. We address their concerns below.

2.1 Comment 2.1: Data deduplication strategy justification

Reviewer Comment: I was surprised by the following line of the paper: "Data deduplication. Since existing datasets often contain significant structural redundancy, we also experiment with two data deduplication strategies – we present these in more detail in Appendix A. For the remainder of this paper, however, we make use of the non-deduplicated version of SPINDR." This choice seems surprising, and more discussion in the main text of the paper is warranted. A clear justification for the choice was also not apparent to this reader after reviewing the material in the Appendix. Some additional clarity around this methodological choice in the main text of the manuscript would benefit the reader.

Our Response:
We conducted experiments across all three dataset versions: non-deduplicated, RMSD-based deduplication, and combined RMSD-sequence identity deduplication. Evaluating ligand generation performance on the same holdout test set, we found no significant performance improvement with RMSD-based deduplication (in fact, slightly worse), while RMSD-sequence identity deduplication led to notably degraded results. We attribute this decline primarily to reduced dataset size: RMSD-sequence identity deduplication removes ∼15,000 complexes, while RMSD-only removes ∼10,000 complexes.
Given that deduplication provided no clear performance benefits while substantially reducing training data—particularly problematic for learning the complex chemical and conformational distributions required for this task—we opted to use the full non-deduplicated dataset. However, a recent study focusing on binding affinity prediction has found that dataset deduplication can play a significant role in generalization. Therefore, we have open-sourced all three dataset versions to enable the community to further investigate the impact of deduplication strategies specifically in the context of ligand generation tasks.
Changes to manuscript: We added a clarification of this rationale to the main text.

2.2 Comment 2.2: Strain energy analysis and physical realism

Reviewer Comment: The authors rightly note that the strain energies of generated ligands from both FLOWR (90.05 kcal/mol) and PILOT (120.10 kcal/mol) are substantially higher than that of the test set (43.27 kcal/mol). While they hypothesize this is due to limited training data coverage, this is a significant limitation regarding the physical realism of the generated molecules. This point warrants more discussion. This reviewer could not get a clear sense from the text regarding whether or not the molecules looked 'mangled' versus what one would expect to see from an experimental crystal structure, or even a high-quality docked pose using traditional physics-based docking methods. Would these strained molecules be easily relaxed using local minimization with a classical force field? Exploring these topics in greater detail would be beneficial to the work.

Our Response: We emphasize that high PoseBusters validity scores indicate that generated ligands are not "mangled" - PoseBusters explicitly evaluates among other things double bond flatness, bond angles, bond lengths, internal energy violations, and steric clashes, all of which pass validation at high rates. Additionally, visual inspection of generated structures (shown in our figures on several targets without cherry-picking or any form of post-processing) confirms physically reasonable geometries.

The elevated strain energies however might reflect subtle deviations in bond angles and lengths that incur disproportionate energy penalties in force field evaluations, rather than gross structural defects. To validate this interpretation, we performed simple MMFF94s-based relaxation (via RDKit) on all generated ligands across test set targets with protein pockets being fixed. Post-relaxation, strain energies decrease dramatically to 28.05 ± 28.25 kcal/mol (mean reduction of 62.75 kcal/mol) - notably lower than the test set average of 43.27 ± 41.85 kcal/mol - with a mean RMSD of 0.775 ± 0.144 Å, while mean PB-validity increases to 0.95 ± 0.08 and the mean Vina score decreases to -6.97 ± 0.89. This demonstrates that generated molecules readily relax to low-energy conformations with minimal structural perturbation, confirming their physical plausibility.

These results indicate that FLOWR generates geometrically sound binding poses with only minor force field strain that is correctable through standard minimization protocols.

Changes to manuscript: We added those results to the main text and added Fig. 15 to the Appendix D.3 (Additional Experimental Results).

We believe these revisions have substantially strengthened the manuscript and addressed all concerns raised by the reviewers. We hope the revised manuscript is now suitable for publication in *Nature Computational Science*.

Sincerely,

Julian Cremer and Ross Irwin (on behalf of all authors)
